# Evidence-based recommender system for high-entropy alloys

**DOI:** 10.1038/s43588-021-00097-w

**Published:** 2021-07-19

**Authors:** Minh-Quyet Ha, Duong-Nguyen Nguyen, Viet-Cuong Nguyen, Takahiro Nagata, Toyohiro Chikyow, Hiori Kino, Takashi Miyake, Thierry Denœux, Van-Nam Huynh, Hieu-Chi Dam

**Affiliations:** 1 Japan Advanced Institute of Science and Technology, Nomi, Japan; 2 HPC SYSTEMS Inc., Tokyo, Japan; 3 Research Center for Functional Materials, National Institute for Materials Science, Tsukuba, Japan; 4 Research and Services Division of Materials Data and Integrated System, National Institute for Materials Science, Tsukuba, Japan; 5 Research Center for Computational Design of Advanced Functional Materials, National Institute of Advanced Industrial Science and Technology, Tsukuba, Japan; 6 Université de technologie de Compiègne, CNRS, UMR 7253 Heudiasyc, Compiègne, France

**Keywords:** Computational methods, Metals and alloys, Information theory and computation

## Abstract

Existing data-driven approaches for exploring high-entropy alloys (HEAs) face three challenges: numerous element-combination candidates, designing appropriate descriptors, and limited and biased existing data. To overcome these issues, here we show the development of an evidence-based material recommender system (ERS) that adopts Dempster–Shafer theory, a general framework for reasoning with uncertainty. Herein, without using material descriptors, we model, collect and combine pieces of evidence from data about the HEA phase existence of alloys. To evaluate the ERS, we compared its HEA-recommendation capability with those of matrix-factorization- and supervised-learning-based recommender systems on four widely known datasets of up-to-five-component alloys. The *k*-fold cross-validation on the datasets suggests that the ERS outperforms all competitors. Furthermore, the ERS shows good extrapolation capabilities in recommending quaternary and quinary HEAs. We experimentally validated the most strongly recommended Fe–Co-based magnetic HEA (namely, FeCoMnNi) and confirmed that its thin film shows a body-centered cubic structure.

## Abstract (web summary)

An evidence-based approach for dealing with insufficient, conflicting and biased materials data is proposed for recommending high-entropy alloys, showing good capabilities for extrapolating the number of components.

## Main

Multiprinciple element alloys (MPEAs, among which alloys with ≥5 elements are also called high-entropy alloys, HEAs) are a new alloy development concept^1–3^ that comprise multiple elements and form highly disordered solid-solution phases. Since their discovery, MPEAs and HEAs have attracted the interest of the scientific community owing to their promising properties and potential applications^4,5^. Such alloys show high strength-to-weight ratios, tensile strengths, and corrosion and oxidation resistances. For consistency with the published data used in this study, we use the term HEA to refer to random alloys comprising multiple equiatomically combined elements and that form a solid-solution phase. From the materials development perspective, specific element combinations that will most likely form single-phase HEAs must necessarily be recommended for experimental validation. Deductive and inductive approaches are used to accomplish this task, and are based on entirely different concepts.

In the deductive approach, it is necessary to understand the HEA formation mechanisms or begin with the quantum-mechanics equations derived on the basis of numerous first-principles calculations. In previous HEA research, it was hypothesized that HEA constituent elements form a single-phase solid solution owing to configurational-entropy-induced stabilization; however, this hypothesis is correct only for some multicomponent alloys, most of which have been experimentally demonstrated to form multiple phases^6^. Although much attention has been devoted to the formation mechanism driving HEA stability, the key factors governing the formation of single-phase HEAs remain unknown^7^. Constructing phase diagrams for multicomponent alloys by first-principles calculations can also directly predict which alloys will form solid solutions^8^; however, this method involves energy calculations for many configurations and the implementation of statistical mechanical models for estimating thermodynamic properties, both of which are computationally demanding^9^. It is therefore imperative to search for HEAs by first-principles calculations.

Several inductive screening methods have been developed using descriptors created from knowledge of condensed matter theory, with parameters fitted to the available experimental data to predict the possible HEAs^10,11^ or their structure phases^12–14^; however, applying the inductive approach requires sufficient and balanced data to ensure prediction accuracy; these are usually not available with experimental material data, which are either lacking or heavily biased towards positive results^15,16^. Furthermore, although it would be desirable to quantitatively evaluate the prediction uncertainty even if a high prediction accuracy cannot be obtained, this has not yet been achieved. Another challenge is to design suitable material descriptors to represent the data of alloys comprising different numbers of elements. Descriptors calculated from the atomic properties of the constituent elements (for example, mean, variance and difference of atomic sizes) are often adopted^14,17–21^; however, it is mathematically difficult to accurately assess the similarities or dissimilarities between alloys with different numbers of compositions, and there are inevitable limits to the results obtained by data-driven approaches using these descriptors^17,22^. A solution to this problem is to describe the alloy using one-hot vectors of constituent elements; however, this approach raises another difficulty, which is designing a proper metric in this vector space^23^.

To overcome these issues and focus on predicting whether the HEA phase exists for particular combinations of elements, we adopted the Dempster–Shafer theory^24–26^ (referred to as the evidence theory) to develop a descriptor-free recommender system named the evidence-based recommender system (ERS) for exploring potential HEAs.

The Dempster–Shafer theory can be considered as a generalization of the Bayesian approach for dealing with situations of incomplete information and imperfect data, and is deemed suitable for solving material data problems. Given a set (*Ω*) of possibilities (called the frame of discernment), evidence theory assigns non-negative weights (summing to one) to subsets of *Ω*, instead of assigning them to elements of *Ω* as in the Bayesian approach. By adopting the evidence theory, we can model, collect and combine pieces of evidence from multiple alloy data without using material descriptors. Consequently, the proposed system can suggest HEAs by learning from multiple data of alloys with fewer constituent elements.

The proposed ERS is based on the elemental substitution method widely used to synthesize various materials. This method is used to replace the element or group of elements with a counterpart showing similar chemical functions, such that the properties of the target material are not affected. The difficulty in this approach is the proper assessment of the similarity between the chemical functions of the alloy metal combinations to discover potential HEAs. To address this issue, we consider each pair of observed alloys as a piece of evidence to compare the contribution of their constituent elements or a combination thereof to the target property (forming HEA phase). The obtained similarity evidence is then used to generate evidence for hypothesizing whether the substituted alloys are HEAs. The ERS consists of three main steps (Supplementary Fig. 1):

1. Measure the similarity between element combinations: all of the pieces of evidence obtained from the data are modeled and combined to conclude the similarity between the element combinations by using evidence theory.
2. Evaluate the hypothesis on the properties of the substituted alloys: the pieces of evidence for the substituted alloys are modeled and combined to evaluate the hypothesis about the target property (forming HEA phase) by using evidence theory.
3. Rank substituted alloys: the substituted alloys are ranked according to various criteria based on the combined evidence of their target properties to recommend potential HEAs.

## Results

### ERS methodology

Each alloy *A* in dataset ${\mathcal{D}}$ is represented by a set of its components. The property of interest *y*_*A*_ for *A*, which can either be HEA or not HEA (¬HEA), indicates whether the HEA phase exists for *A*. We first measure the similarity between element combinations by adapting the evidence theory to model and combine all pieces of evidence obtained from ${\mathcal{D}}$.

**Fig. 1:**
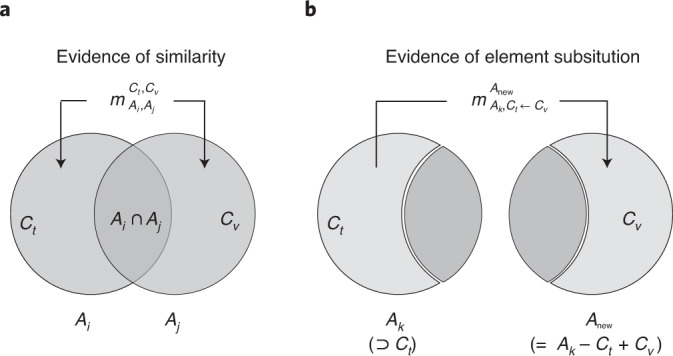
An illustration of the ERS methodology. **a**,**b**, Venn diagrams shows the logical relationships between alloys (*A*_*i*_, *A*_*j*_, *A*_*k*_ and *A*_new_) and element combinations (*C*_*t*_ and *C*_*v*_), which are used to model evidence of similarities between element combinations (**a**) and new alloys by the element-substitution method (**b**).

Similarities between objects appear in various forms^27^: ratings of pairs, sortings of objects, communality betweeen associations, substitutability, and correlation between occurrences. Here the solid-solution formability for combinations of elements are discussed, along with the measure of similarity in terms of substitutability between the combinations of elements. Each non-disjoint pair of alloys *A*_*i*_ and *A*_*j*_ in ${\mathcal{D}}$ is a source of evidence for measuring the substitutability between element combinations *C*_*t*_ = *A*_*i*_ − (*A*_*i*_ ∩ *A*_*j*_) = *A*_*i*_ − *A*_*j*_ and *C*_*v*_ = *A*_*j*_ − (*A*_*i*_ ∩ *A*_*j*_) = *A*_*j*_ − *A*_*i*_ (Fig. 1a). The non-empty intersection set *A*_*i*_ ∩ *A*_*j*_ is considered as the context for the similarity measurement. If ${y}_{{A}_{i}}={y}_{{A}_{j}}$ then *C*_*t*_ and *C*_*v*_ are substitutable, otherwise *C*_*t*_ and *C*_*v*_ are not substitutable.

To model evidence about the similarity between any pair of element combinations, we first define a frame of discernment^25^, *Ω*_sim_ = {similar, dissimilar} containing all possible values. The evidence collected from alloys *A*_*i*_ and *A*_*j*_ is then represented by a mass function^25^ (or a basic probability assignment), ${m}_{{A}_{i},{A}_{j}}^{{C}_{t},{C}_{v}}$, which assigns probability masses to all of the non-empty subsets of *Ω*_sim_ (that is, {similar}, {dissimilar} and {similar, dissimilar}), as follows:

1 $${m}_{{A}_{i},{A}_{j}}^{{C}_{t},{C}_{v}}(\{\mathrm{similar}\})=\left\{\begin{array}{ll}\alpha &\,\text{if}\,{y}_{{A}_{i}}={y}_{{A}_{j}}\\ 0&\,\text{otherwise}\,\end{array}\right.,$$

2 $${m}_{{A}_{i},{A}_{j}}^{{C}_{t},{C}_{v}}(\{\mathrm{dissimilar}\})=\left\{\begin{array}{ll}\alpha &\,\text{if}\,{y}_{{A}_{i}}\ne {y}_{{A}_{j}}\\ 0&\,\text{otherwise}\,\end{array}\right.,$$

3 $${m}_{{A}_{i},{A}_{j}}^{{C}_{t},{C}_{v}}(\{\mathrm{similar,dissimilar}\})=1-\alpha$$

Note that the masses assigned to {similar} and {dissimilar} indicate the degrees of belief exactly committed to *A*_*i*_ and *A*_*j*_ to support the similarity and dissimilarity between *C*_*t*_ and *C*_*v*_, respectively. The weight assigned to subset {similar, dissimilar} expresses the degree of belief that *A*_*i*_ and *A*_*j*_ provide no information about the similarity (or dissimilarity) between *C*_*t*_ and *C*_*v*_. Here the parameter *α* is determined by an exhaustive search (0 < *α* < 1) for the best cross-validation score (Methods). We retain some degree of uncertainty (1 − *α*) about the similarities collected from each piece of evidence for dealing with the inconsistencies in the dataset. The sum of the masses assigned to all three non-empty subsets of *Ω*_sim_ is 1.

Suppose that we can collect multiple pieces of evidence from ${\mathcal{D}}$ to compare two element combinations *C*_*t*_ and *C*_*v*_, all obtained mass functions corresponding to those pieces of evidence are then combined using the Dempster rule of combinations^24^ to assign the final mass ${m}_{{\mathcal{D}}}^{{C}_{t},{C}_{v}}$ (Methods). Similar analyses are performed for all pairs of element combinations of interest to obtain a symmetric matrix *M* that comprises all of the similarities between them ($M[t,v]=M[v,t]={m}_{{\mathcal{D}}}^{{C}_{t},{C}_{v}}(\{\mathrm{similar}\})$).

For hypothesizing whether a potential alloy *A*_new_ forms an HEA phase, we apply the substitution method using the obtained *M*. We replace a combination of elements, *C*_*t*_, in an existing alloy, *A*_*k*_, (*C*_*t*_ ⊂ *A*_*k*_) with a combination of elements, *C*_*v*_, adequate to obtain alloy *A*_new_, showing a property (label ${y}_{{A}_{\mathrm{new}}}$) similar to that of *A*_*k*_ (label ${y}_{{A}_{k}}$). The basic beliefs on the *A*_new_ label are quantified on the basis of the label of *A*_*k*_ and the similarity between *C*_*t*_ and *C*_*v*_ (Fig. 1b). If *C*_*t*_ and *C*_*v*_ are substitutable (non-substitutable), this serves as a piece of evidence that the labels of *A*_new_ and *A*_*k*_ are the same (different).

To model evidence about the existence of an HEA phase in a particular alloy, we first define a frame of discernment^25^ *Ω*_HEA_ = {HEA, ¬HEA}. The evidence collected from *A*_*k*_, *C*_*t*_ and *C*_*v*_ is then represented by the mass function ${m}_{{A}_{k},{C}_{t}\leftarrow {C}_{v}}^{{A}_{\mathrm{new}}}$, which assigns probability masses to all of the non-empty subsets of Ω_HEA_ (that is, {HEA}, {¬HEA} and {HEA, ¬HEA}), as follows:

4 $${m}_{{A}_{k},{C}_{t}\leftarrow {C}_{v}}^{{A}_{\mathrm{new}}}(\{\mathrm{HEA}\})=\left\{\begin{array}{ll}M[t,v]&\,\text{if}\,{y}_{{A}_{k}}={\mathrm{HEA}}\\ 0&\,\text{otherwise}\,\end{array}\right.,$$

5 $${m}_{{A}_{k},{C}_{t}\leftarrow {C}_{v}}^{{A}_{\mathrm{new}}}(\neg {\mathrm{HEA}})=\left\{\begin{array}{ll}M[t,v]&\,\text{if}\,{y}_{{A}_{k}}= \neg {\mathrm{HEA}}\\ 0&\,\text{otherwise}\,\end{array}\right.,$$

6 $${m}_{{A}_{k},{C}_{t}\leftarrow {C}_{v}}^{{A}_{\mathrm{new}}}({\mathrm{HEA}},\neg {\mathrm{HEA}})=1-M[t,v],$$

Note that the masses assigned to {HEA} and {¬HEA} reflect the levels of confidence, whereby *A*_*k*_ and the substitution of *C*_*v*_ for *C*_*t*_ support the probabilities that *A*_new_ is or is not an HEA, respectively. The mass assigned to subset {HEA, ¬HEA} expresses the probability that *A*_*k*_, *C*_*t*_ and *C*_*v*_ provide no information about the property of *A*_new_. The sum of the probability masses assigned to all three non-empty subsets of *Ω*_HEA_ is 1.

We assume that for a specific hypothetical alloy, *A*_new_, we can collect pieces of evidence about its properties from ${\mathcal{D}}$ (pair of *A*_host_ and the corresponding substitution to obtain *A*_new_ from *A*_host_). The obtained mass functions for *A*_new_ are then combined using the Dempster rule^24^ to obtain a final mass function ${m}^{{A}_{\mathrm{new}}}$ (Methods). Similar analyses are performed for all of the possible alloys (*A*_new_) that are not included in the observed data. We then use the final value of ${m}_{{\mathcal{D}}}^{{A}_{\mathrm{new}}}(\{\mathrm{HEA}\})$ for sorting the ranking of recommendation. The proposed recommender system considers alloys with a higher value of ${m}_{{\mathcal{D}}}^{{A}_{\mathrm{new}}}(\{\mathrm{HEA}\})$ to have greater potential of having HEA phases.

### Experimental settings

We use eight datasets (Table 1) consisting of binary, ternary, quaternary and quinary alloys comprising multiple equiatomically combined elements to evaluate the proposed system for recommending HEAs and revealing the HEA formation mechanisms. The alloys contained in the datasets comprise ${\mathcal{E}}=$ {Fe, Co, Ir, Cu, Ni, Pt, Pd, Rh, Au, Ag, Ru, Os, Si, As, Al, Tc, Re, Mn, Ta, Ti, W, Mo, Cr, V, Hf, Nb and Zr}. Any alloy contained in the following datasets is predicted as an HEA if its order–disorder transition temperature is below its melting temperature. All of the datasets are shown in detail in Supplementary Section 2.

**Table 1:** Summary of alloy datasets used in evaluation experiments

Dataset	No. alloys	No. HEAs	No. candidates		HEA rate	Observation rate
${{\mathcal{D}}}_{\text{ASMI16}}$^48^	45 Binary alloys	45	351		100%	13%
${{\mathcal{D}}}_{\text{CALPHAD}}$^3,49^	243 Ternary alloys	243	2,925		100%	9%
${{\mathcal{D}}}_{\text{AFLOW}}$^50^	117 Binary alloys	60	351		51%	33%
	441 Ternary alloys	234	2,925		53%	15%
${{\mathcal{D}}}_{\text{LTVC}}$^43^	117 Binary alloys	58	351		49%	33%
	441 Ternary alloys	148	2,925		33%	15%
${{\mathcal{D}}}_{\,\text{AFLOW}}^{\text{quaternary}\,}$^50^	1,110 Quaternary alloys	754	17,550		68%	6%
${{\mathcal{D}}}_{\,\text{LTVC}}^{\text{quaternary}\,}$^43^	1,110 Quaternary alloys	480	17,550		43%	6%
${{\mathcal{D}}}_{\,\text{AFLOW}}^{\text{quinary}\,}$^50^	130 Quinary alloys	129	80,730		99%	0.16%
${{\mathcal{D}}}_{\,\text{LTVC}}^{\text{quinary}\,}$^43^	130 Quinary alloys	91	80,730		70%	0.16%

No. alloys, number of alloys included in each dataset; no. HEAs, number of the alloys confirmed or estimated to form HEA phase in each dataset; no. candidates, number of possible alloys generated using the set of all elements in the datasets; HEA rate, the ratio of no. HEA to no. alloys; observation rate, ratio of no. alloys to no. candidates.

It should be noted that our system has the capability to collect and combine evidence from multiple datasets to reasonably draw the final conclusions. However, in the evaluation of HEA-recommendation capability, each dataset comes from a different experiment or calculation method; we therefore evaluate the proposed method with each dataset separately to ensure consistency between the training and test sets.

We compare the HEA-recommendation performance of the proposed ERS with those of matrix-based recommender systems^28^ previously developed using non-negative matrix factorization (NMF)^29^ and singular-value decomposition (SVD)^30^. We apply two types of rating matrix representations to use the matrix-based recommender systems for exploring potential HEAs. Furthermore, the performances of recommender systems that are based on supervised-learning methods (support vector machines^31^ (SVMs), logistic-regression^32^, decision tree^33^ and naive Bayes^34^) are compared with that of the ERS. We apply a compositional descriptor to employ the SVM- and logistic-regression-based recommender systems. The binary elemental descriptor is used to represent the alloys in our system and in the decision-tree- and naive-Bayes-based recommender systems. The material descriptors are shown in detail in the Methods.

### Learning about the similarity between elements

**Fig. 2:**
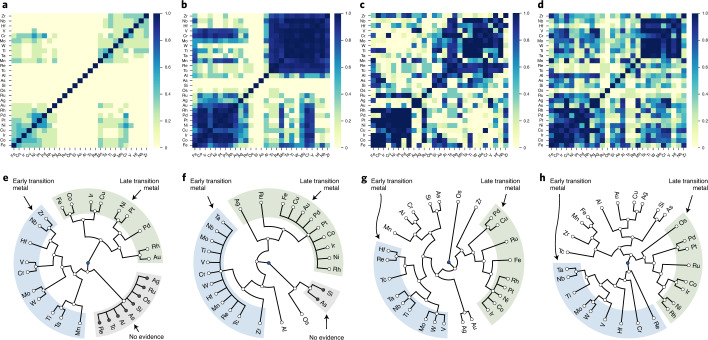
Visualization of similarities between elements. **a**–**d**, Heat maps for similarity matrices *M*_ASMI16_ (**a**), *M*_CALPHAD_ (**b**), *M*_AFLOW_ (**c**) and *M*_LTVC_ (**d**). Each matrix element is the probability mass that the similarity mass function of the corresponding element pair is assigned to subset {similar} of *Ω*_sim_. These matrix elements indicate the degree of belief learned from the similarity data of the corresponding element pairs. The degrees of belief are illustrated using colorscale bars. **e**–**h**, Hierarchically clustered structures of all ${\mathcal{E}}$ constructed using hierarchical agglomerative clustering and the *M*_ASMI16_ (**e**), *M*_CALPHAD_ (**f**), *M*_AFLOW_ (**g**) and *M*_LTVC_ (**h**) datasets. The blue, green and gray regions indicate groups of early and late transition metals, and elements without similarity evidence, respectively. Source data

By applying the proposed ERS to ${{\mathcal{D}}}_{\text{ASMI16}}$, ${{\mathcal{D}}}_{\text{CALPHAD}}$, ${{\mathcal{D}}}_{\text{AFLOW}}$ and ${{\mathcal{D}}}_{\text{LTVC}}$ (Table 1), we assess the similarities between the elements ${\mathcal{E}}$ and all of the possible binary combinations obtained therein. Figure 2a–d shows the *M*_ASMI16_, *M*_CALPHAD_, *M*_AFLOW_ and *M*_LTVC_ similarity matrices obtained for all ${\mathcal{E}}$ in the first four experiments. These similarity matrices are then properly transformed into distance matrices to which Ward’s hierarchical agglomerative clustering^35^ can be applied to construct the corresponding hierarchically clustered structures of these elements (Fig. 2e–h).

The similarity matrix *M*_ASMI16_ reveals three distinct element groups (Fig. 2e) consisting of Ti, V, Cr, Mn, Zr, Nb, Mo, Hf, Ta and W; Fe, Co, Ni, Cu, Rh, Pd, Ir, Pt and Au; and Al, Ag, Tc, Si, Ru, As, Re and Os, where the first two groups correspond to the early and late transition metals, respectively. Given the similar physical and chemical properties of these elements, the high degree of similarity between the elements within the same group is rational, as revealed by the ERS. Interestingly, *M*_ASMI16_ shows a remarkable similarity between manganese (an earlier transition metal) and gold (a late transition metal). Furthermore, the *M*_ASMI16_ indicates none of the belief about the similarities among the elements in the third group, and between the elements of the third group and the other two groups as the binary alloys contained in ${{\mathcal{D}}}_{\text{ASMI16}}$ do not contain these elements (Supplementary Fig. 2a). No evidence of similarities can therefore be collected from ${{\mathcal{D}}}_{\text{ASMI16}}$ for these elements.

The similarity matrix *M*_CALPHAD_ also reveals three somewhat modified element groups (Fig. 2f) compared with those obtained from ${{\mathcal{D}}}_{\text{ASMI16}}$. As ${{\mathcal{D}}}_{\text{CALPHAD}}$ contains some technetium- and rhenium-containing alloys, these elements join the group of early transition metals. Similarly, ${{\mathcal{D}}}_{\text{CALPHAD}}$ contains more silver- and ruthenium-containing alloys, and these elements join the group of late transition metals; thus, only aluminum, silicon, astatine and osmium remain in the third group. Although no evidence of any similarities between silicon and astatine can be collected from ${{\mathcal{D}}}_{\text{CALPHAD}}$ (Supplementary Fig. 2a), osmium and aluminum are somewhat similar to the first and second groups, respectively.

By contrast, it is difficult to divide all of the elements contained in ${\mathcal{E}}$ into groups according to the matrix *M*_AFLOW_; however, some characteristic groups of metallic elements are distinct. Although two distinct groups of early or late transition metals are observed (Fig. 2g), there are some notable differences between these results and those obtained from ${{\mathcal{D}}}_{\text{ASMI16}}$ and ${{\mathcal{D}}}_{\text{CALPHAD}}$ (Supplementary Section 3). Furthermore, the similarity matrix *M*_AFLOW_ does not show any similarity between osmium and any of the other elements as very few osmium-containing alloys are contained in the dataset (Supplementary Fig. 2a). Furthermore, the similarity matrices *M*_LTVC_ and *M*_AFLOW_ are approximately similar; however, the hierarchically clustered structure constructed from ${{\mathcal{D}}}_{\text{LTVC}}$ indicates that copper, silver and gold form a distinct subgroup (Fig. 2h).

**Fig. 3:**
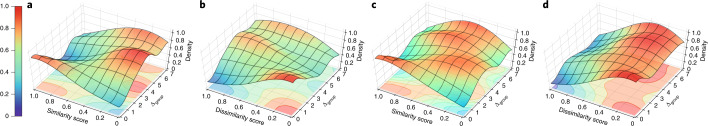
Correlation between pairwise similarity scores and Δ_group_ of elements. **a**–**d**, Subfigures illustrate the distribution of pairwise similarities, which are obtained from ${{\mathcal{D}}}_{\text{AFLOW}}$ (**a**,**b**) and ${{\mathcal{D}}}_{\text{LTVC}}$ (**c**,**d**) according to the Δ_group_ of these element pairs. The colorscale bar illustrates the estimated density of the distribution of pairwise similarities. Source data

Figure 3 shows the correlation between the pairwise similarity scores learned from ${\mathcal{D}}_{\mathrm{AFLOW}}$ and ${\mathcal{D}}_{\mathrm{LTVC}}$, and the corresponding difference between the periodic table group index (Δ_group_) obtained for each of the transition metal pairs contained in ${\mathcal{E}}$. The elements showing the same periodic table group index (Δ_group_ = 0) clearly tend to show high similarity scores (Fig. 3a,c) and low dissimilarity scores (Fig. 3b,d). The elements in the same group thus similarly contribute to HEA formation and are substitutable for each other; however, it should be noted that several pairs of elements have a similarity with a low degree of belief despite them belonging to the same groups, that is {(Ti, Zr), (Cu, Ag), (Fe, Ru)} in ${{\mathcal{D}}}_{\text{AFLOW}}$ and {(Ti, Zr), (Mn, Re), (Ni, Pd)} in ${{\mathcal{D}}}_{\text{LTVC}}$ (Fig. 2c,d).

Furthermore, as Δ_group_ increases from 0 to 4, similarity scores between the elements decrease. The results learned from both ${{\mathcal{D}}}_{\text{AFLOW}}$ and ${{\mathcal{D}}}_{\text{LTVC}}$ show that the elements are the least similar when the difference between their group indices is three or four; however, the elements become slightly more similar as Δ_group_ increases from 5 to 7, which is consistent with the domain knowledge about the differences between early and late transition metals.

### Evaluation of recommendation capability by cross-validation

We apply *k*-fold cross-validation to ${{\mathcal{D}}}_{\text{ASMI16}}$, ${{\mathcal{D}}}_{\text{CALPHAD}}$, ${{\mathcal{D}}}_{\text{AFLOW}}$ and ${{\mathcal{D}}}_{\text{LTVC}}$ to assess the HEA-recommendation capabilities of the ERS and four matrix-based recommender systems (NMF and SVD, each one with two types of matrix representations)^28^. These two matrix representations (types 1 and 2) decompose an alloy into two elementary components *A* and *B* with different sizes (Methods). We also compare the ERS with the four supervised-learning-method-based (decision tree, naive Bayes, logistic-regression and SVM) recommender systems.

The learned similarity matrix is used to rank all of the alloys contained in the test set and all of the possible combinatorial alloys other than those used to train the similarity matrix. The resulting alloy rankings are then used to evaluate the HEA-recommendation performance. We designed a virtual experiment that sequentially identifies the alloys on the basis of the order in which they were previously ranked. To evaluate the HEA-recommendation capability of the proposed ERS, we monitor the rank of HEAs in the test set and the HEA recall depending on the number of trials required to identify all possible HEAs. Detailed experimental conditions are shown in the Methods.

**Fig. 4:**
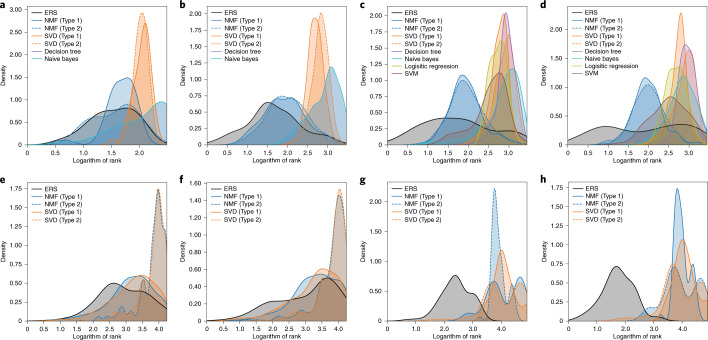
Evaluation of HEA-recommendation capability. Probability density functions of the rank of the HEAs in the test sets in the ${{\mathcal{D}}}_{\text{ASMI16}}$ (**a**), ${{\mathcal{D}}}_{\text{CALPHAD}}$ (**b**), ${{\mathcal{D}}}_{\text{AFLOW}}$ (**c**), ${{\mathcal{D}}}_{\text{LTVC}}$ (**d**), ${{\mathcal{D}}}_{\,\text{AFLOW}}^{\text{quaternary}\,}$ (**e**), ${{\mathcal{D}}}_{\,\text{LTVC}}^{\text{quaternary}\,}$ (**f**), ${{\mathcal{D}}}_{\,\text{AFLOW}}^{\text{quinary}\,}$ (**g**) and ${{\mathcal{D}}}_{\,\text{LTVC}}^{\text{quinary}\,}$ (**h**) experiments. The ranks of HEAs in the test sets are expressed on a base-10 logarithmic scale. The HEAs with higher ranking order are recommended materials with a firmer belief in the formation of the HEA phase. Source data

Figure 4a–d illustrates the distributions of the HEA ranks of the test set recommended by the different systems. The HEAs in the test set are generally recommended with higher rank using the ERS (that is, the ERS rank distributions are on the left of the curves for the other systems). Consequently, the ERS can considerably reduce the number of trials required to recover the HEAs in the test set compared with the competitor systems. Only in the experiment with ${{\mathcal{D}}}_{\text{ASMI16}}$ are the distributions of the rank using the ERS and NMF (type 2) somewhat similar (Fig. 4a). We also monitor the dependence of the HEA recall ratio on the number of trials required to measure the HEA-recommendation performance of the ERS quantitatively. In summary, the ERS outperforms the other systems in recalling one-half and three-quarters of the HEAs in the test set (Supplementary Section 4A); however, the ERS cannot reliably recall the remaining one-quarter of the HEAs as insufficient evidence is available in the training data to make inferences about the remaining HEAs. Interestingly, in the ${{\mathcal{D}}}_{\text{ASMI16}}$ and ${{\mathcal{D}}}_{\text{CALPHAD}}$ experiments, the supervised-method-based recommender systems either approximately randomly selected possible HEAs (naive Bayes and decision tree) or could not rank any at all (logistic regression and SVM) as these datasets contain only positively labeled HEAs.

### Evaluation of recommendation capability by extrapolation

The cross-validation experiments show that the recommendation systems based on supervised learning methods (SVMs^31^, logistic regression^32^, decision trees^33^ and naive Bayes^34^) have much lower recommendation performance. These results are attributed to the inappropriate assessment of the similarity scores between alloys with different numbers of compositions (Methods); thus, to evaluate the HEA-recommendation capability when extrapolating the number of components, we focus on comparing the performances of the ERS with those of matrix-based recommender systems. The detailed experimental settings are shown in the Methods.

Figure 4e–h illustrates the distributions of the recommended HEA rank of the quaternary and quinary HEAs in the test set that are extrapolated using recommender systems. The obtained results show that the ERS outperforms the capability of the competitor systems at recommending quaternary HEAs (Fig. 4e,f) and substantially outperforms the capability of the other systems at recommending quinary HEAs (Fig. 4g,h). Interestingly, in the experiments with ${{\mathcal{D}}}_{\,\text{LTVC}}^{\text{quinary}\,}$ and ${{\mathcal{D}}}_{\,\text{AFLOW}}^{\text{quinary}\,}$, the numbers of quinary HEAs in the test set—and those found in the top-100 and top-1,000 HEA candidates recommended by the ERS—are much larger than those predicted by the competitor systems. These numbers are very high as the two datasets only contain quinary alloys of the early transition metals. Much of the evidence of the similarities between these element combinations can be collected from the corresponding datasets containing binary, ternary and quaternary alloys (Supplementary Fig. 2b). Moreover, to recall 50 and 75% of the quinary HEAs from these datasets, approximately 10–100 fewer trials are required by the ERS than by the NMF- and SVD-based recommender systems. The results of experiments monitoring the dependence of the HEA recall ratio on the number of trials required are listed in detail in Supplementary Section 4B. In the absence of sufficient evidence, the answer of the system, regarding a mixture of many types of elements, will retain a large degree of uncertainty (*m*({HEA, ¬HEA}) ≈ 1).

### Synthesis of recommended FeMnCo-based HEAs

Fe–Co-based film soft-magnetic materials have attracted interest from device community and will be applied to improve the performance of next-generation high-power devices^36^. We therefore focus on Fe–Co-based quaternary alloys containing the first transition-series elements. We combine all evidence collected from all of the datasets to recommend quaternary Fe–Co-based HEAs for experimental validation.

**Fig. 5:**
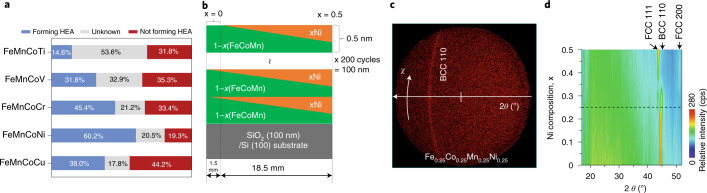
Recommendation and experimental validation for thin film of FeCoMnNi HEA. **a**, Recommended candidates for Fe–Co-based HEAs containing first-transition-series elements: FeMnCoTi, FeMnCoV, FeMnCoCr, FeMnCoNi and FeMnCoCu. **b**, A schematic illustration of the sample—which includes 200 cycles of 0.5 nm spread film—fabricated on SiO_2_/Si(100) substrate using the combinatorial method. Each spread film consists of a 0.25 nm FeCoMn sublayer and a 0.25 nm 1-*x*(FeCoMn)–*x*Ni sublayer. **c**, A two-dimensional X-ray diffraction image of a Fe_0.25_Co_0.25_Mn_0.25_Ni_0.25_ thin film measured by changing the angle *χ* of the incident X-rays. **d**, A heat map showing the dependence of the X-ray diffraction intensity of 1-*x*(FeCoMn)–*x*Ni films on nickel composition and diffraction angle *θ*. Source data

Figure 5a shows the recommended possible magnetic quaternary HEAs containing iron, manganese and cobalt. FeMnCoNi is clearly the only HEA candidate recommended with a belief higher than 0.5. Although FeMnCoCr and FeMnCoCu are HEA candidates recommended with the next highest beliefs, some uncertainty still remains as to their potential as HEAs. We thus chose FeMnCoNi as the target HEA candidate for the experimental validation (see Fig. 5 and the Methods for further information).

Figure 5c shows a two-dimensional X-ray diffraction (XRD) image of a region of the Fe_0.25_Co_0.25_Mn_0.25_Ni_0.25_ alloy annealed at 400 °C. A reflection attributed to the (110) plane of the BCC crystal structure appears in the ring pattern at 2*θ* = 44.7° (PDF 03-065-7519; ref. ^37^). Note that out-of-plane XRD measurements were also performed to identify the crystal structure in more detail, as shown in Supplementary Fig. 6a, indicating the formation of a polycrystalline film. The BCC crystal structure of the FeCoMn alloy is reportedly stable^38^ and previous reports have mentioned that FeCoMnNi alloy has a face-centered cubic FCC structure in high-temperature synthesized bulk; however, detailed information is still not available^39,40^. To investigate the stability of the crystal structure, the effect of nickel doping on the crystal structure was therefore analyzed based on the heat map generated from the X-ray diffraction patterns of FeCoMn films prepared with various nickel contents (Fig. 5d). For a nickel content above 0.3, the FCC structure is also observed at 2*θ* = 43.5°, corresponding to the (111) reflection (Supplementary Fig. 6b) (PDF 03-065-5131; ref. ^37^). These results suggest that the Fe_0.25_Co_0.25_Mn_0.25_Ni_0.25_ HEA shows a BCC structure. In our experiment, the BCC structure of the starting material, FeCoMn, is considered as an essential reason for which the thin films produced by this method tend to be in the BCC phase.

## Discussion

Application of inductive approach usually requires sufficient and balanced data to ensure prediction accuracy; however, material data are usually lacking or heavily biased toward positive results (Table 1). It is very challenging to build prediction models using such small data and a very heavy skew towards positive results. Conflicts within and between datasets of materials are also challenges that inductive approaches must overcome. Quantitative assessment of the uncertainty of the prediction itself is thus indispensable. The ERS has the advantage in dealing with these situations. Instead of forcibly merging data from multiple datasets, our system rationally considers each dataset as a source of evidence and combines the evidence to reasonably draw the final conclusions for recommending HEA, where the uncertainty can be quantitatively evaluated.

To serve the purpose of screening the elements combination forming HEA phases, the ERS focuses on the fundamental question of whether the HEA phase exists. We design a frame of discernment *Ω*_HEA_ = {HEA, ¬HEA} to model the existence of HEA phases with mass functions. Consequently, the ERS has not answered essential questions regarding the structure and other properties of the HEAs; however, by redesigning the frame of discernment reflecting the additional properties of interest, we can also construct a model that can recommend the potential alloys forming the HEA phases with the desirable properties. Furthermore, in the experimental validation, detailed quantitative investigation of the secondary phases in the synthesized FeCoMnNi alloy thin film was not done due to technical difficulties.

## Methods

### Combining multiple pieces of evidence

We assume that we can collect *q* pieces of evidence from ${\mathcal{D}}$ to compare a specific pair of element combinations, *C*_*t*_ and *C*_*v*_. If no evidence is found, the mass function ${m}_{\mathrm{none}}^{{C}_{t},{C}_{v}}$ is initialized, which assigns a probability mass of 1 to the subset {similar, dissimilar}; ${m}_{\mathrm{none}}^{{C}_{t},{C}_{v}}$ models the condition under which no information about the similarity (or dissimilarity) between *C*_*t*_ and *C*_*v*_ is available. Any two pieces of evidence *a* and *b* modeled by the corresponding mass functions ${m}_{a}^{{C}_{t},{C}_{v}}$ and ${m}_{b}^{{C}_{t},{C}_{v}}$ can be combined using the Dempster rule^24^ to assign the joint mass ${m}_{a,b}^{{C}_{t},{C}_{v}}$ to each subset *ω* of *Ω*_sim_ (that is {similar}, {dissimilar} or {similar, dissimilar}) as follows:

7 $$\begin{array}{lll}{m}_{a,b}^{{C}_{t},{C}_{v}}(\omega )&=&\left({m}_{a}^{{C}_{t},{C}_{v}}\oplus {m}_{b}^{{C}_{t},{C}_{v}}\right)(\omega )\\ &=&\frac{\mathop{\sum}\limits_{\forall {\omega }_{k}\cap {\omega }_{h}=\omega }{m}_{a}^{{C}_{t},{C}_{v}}({\omega }_{k})\times {m}_{b}^{{C}_{t},{C}_{v}}({\omega }_{h})}{1-\mathop{\sum}\limits_{\forall {\omega }_{k}\cap {\omega }_{h}={{\emptyset}}}{m}_{a}^{{C}_{t},{C}_{v}}({\omega }_{k})\times {m}_{b}^{{C}_{t},{C}_{v}}({\omega }_{h})},\end{array}$$

where *ω*, *ω*_*k*_ and *ω*_*h*_ are subsets of Ω_sim_. Note that the Dempster rule is commutative and yields the same result by changing the order of ${m}_{a}^{{C}_{t},{C}_{v}}$ and ${m}_{b}^{{C}_{t},{C}_{v}}$. All of the *q* obtained mass functions corresponding to the *q* collected pieces of evidence from ${\mathcal{D}}$ are then combined using the Dempster rule to assign the final mass ${m}_{{\mathcal{D}}}^{{C}_{t},{C}_{v}}$ as follows:

8 $${m}_{{\mathcal{D}}}^{{C}_{t},{C}_{v}}(\omega )=\left({m}_{1}^{{C}_{t},{C}_{v}}\oplus {m}_{2}^{{C}_{t},{C}_{v}}\oplus \cdots \oplus {m}_{q}^{{C}_{t},{C}_{v}}\right)(\omega ).$$

Multiple pieces of evidence about the label of each new alloy are combined using the similar manner. We assume that for a specific hypothetical alloy, *A*_new_, we can collect pieces of evidence about its properties from ${\mathcal{D}}$ (pair of *A*_host_ and the corresponding substitution to obtain *A*_new_ from *A*_host_). If no evidence is found, ${m}_{\mathrm{none}}^{{A}_{\mathrm{new}}}$ is initialized and a probability mass of 1 is applied to set {HEA, ¬HEA}; ${m}_{\mathrm{none}}^{{A}_{\mathrm{new}}}$ models the condition that no information about the label of *A*_new_ can be obtained from ${\mathcal{D}}$. The obtained mass functions for *A*_new_ are then combined using the Dempster rule^24^ to obtain a final mass function ${m}_{{\mathcal{D}}}^{{A}_{\mathrm{new}}}$ on Ω_HEA_.

### Materials descriptors

Descriptors, which are the representation of alloys, play a crucial role in building a recommender system to explore potential new HEAs. In this research, the raw data of alloys is represented in the form of elements combination. Several descriptors have been studied in materials informatics to represent the compounds^41^. To employ the data-driven approaches for this work, we applied compositional descriptor^20^, rating matrix representation^28^ and binary elemental descriptor^41^.

Compositional descriptor represents an alloy by a set of 135 features composed of means, standard deviations and covariance of established atomic representations that form the alloy. The descriptor can be applied not only to crystalline systems but also to molecular system. We adopted 15 atomic representations: (1) atomic number, (2) atomic mass, (3) period and (4) group in the periodic table, (5) first ionization energy, (6) second ionization energy, (7) Pauling electronegativity, (8) Allen electronegativity, (9) van der Waals radius, (10) covalent radius, (11) atomic radius, (12) melting point, (13) boiling point, (14) density and (15) specific heat; however, the compositional descriptor hardly distinguishes compounds that have different numbers of the atom as it is to regard the atomic representations of a compound as distributions of data. The compositional descriptor therefore cannot be applied in the case of having extrapolation in the number of components.

The rating matrix representation, which is a descriptor-free approach, shows a robust performance of recommendations for a wide variety of datasets in the machine learning community. Seko and colleagues adopted the representation to build a recommender system for exploring currently unknown chemically relevant compositions^28^. In that work, a composition dataset needs to be transformed into just two feature sets, which corresponds to users and items in a user-item rating matrix. Ratings of missing elements are approximately predicted based on the similarity of features given by the representation. To build a recommender system for HEA, we first define the candidate alloys as AB, where *A* and *B* correspond to elementary components of the alloys. We introduce two kinds of matrix representations for the eight alloys datasets. An alloy is decomposed into two elementary components with the following number of elements.

- Type 1: ∣*A*∣ ∈ {1, 2} and ∣*B*∣ ∈ {1, 2, 3}. The numbers of possible components *A* and *B* are 378 and 3,303, respectively. The size of the rating matrix is (378 × 3,303).
- Type 2: ∣*A*∣ = 1 and ∣*B*∣ ∈ {1, 2, 3, 4}. The numbers of possible components *A* and *B* are 27 and 20,853, respectively. The size of the rating matrix is (27 × 20,853).

Binary elemental descriptors is simply a binary digit representing the presence of chemical elements. The number of binary elemental descriptors corresponds to the number of element types included in the training data. In this work, the alloys datasets are composed of 27 kinds of elements; thus, an alloy is described by a 27-dimensional binary vector with elements of one or zero.

### Tuning hyperparameter of the ERS

As the datasets used in this work are the output of calculation prediction methods, we add some degree of uncertainty *α* in the mass function, which models similarity evidence. In each dataset, we use grid search to determine the *α* that best reproduced the alloy labels in the dataset (achieving best cross-validation score). Details of the cross-validation schemes are mentioned in the Methods. The search space of *α* is from 0.01 to 0.9 with a step of 0.01; however, the relative magnitudes of (degree of belief HEA) and (degree of belief not HEA) are almost unchanged. In summary, the absolute value of alpha has little effect on the final result of the recommender system.

### Experimental settings for cross-validation

Cross-validated testing accuracy rates of our method when considered as a supervised learning method are 80% and 75% in ${{\mathcal{D}}}_{\text{AFLOW}}$ and ${{\mathcal{D}}}_{\text{LTVC}}$, respectively, which are almost at the same level with those in the previous study^14^; however, our work pays more attention towards calculating the recall, which is the percentage of the total HEAs correctly classified. This recall value is a more appropriate evaluation measure compared with supervised learning accuracy for finding new combinations of elements having HEA phases.

As ${{\mathcal{D}}}_{\text{ASMI16}}$ only contains binary alloys, we can learn a similarity matrix between the elements from a training set sampled from ${{\mathcal{D}}}_{\text{ASMI16}}$. By applying the proposed process for recommending substituted alloys, we can rank all of the possible binary alloys other than those in the training set. A total of 351 hypothetical binary alloys showing equivalent components can be generated from the 27 elements in ${\mathcal{E}}$, 45 of which are contained in ${{\mathcal{D}}}_{\text{ASMI16}}$. As no information is available for the other 306 alloys, they are ranked by the constructed model. We apply ninefold cross-validation to ${{\mathcal{D}}}_{\text{ASMI16}}$. A total of 40 out of the 45 alloys in ${{\mathcal{D}}}_{\text{ASMI16}}$ are used as the training set, and the remaining five alloys are used as the test set to evaluate the HEA recall rate. The model learned from the 40 alloys in the training set is then used to rank the other 311 alloys, including the five in the test set. This cross-validation is repeated 100 times so that the HEA-recommendation performance can be reliably calculated.

As ${{\mathcal{D}}}_{\text{CALPHAD}}$ only contains ternary alloys, we can learn a similarity matrix between the elements or binary combinations thereof from a training set sampled from ${{\mathcal{D}}}_{\text{CALPHAD}}$. We can build a model to rank all of the possible ternary alloys other than those in the training set. There are 2,925 hypothetical ternary alloys showing equivalent components that can be generated from the 27 elements in ${\mathcal{E}}$, 243 of which are contained in ${{\mathcal{D}}}_{\text{CALPHAD}}$. As no information is available for the other 2,682 alloys, they are ranked by the constructed model. We apply ninefold cross-validation to ${{\mathcal{D}}}_{\text{CALPHAD}}$ and use 216 of the 243 alloys in ${{\mathcal{D}}}_{\text{CALPHAD}}$ as the training set. The remaining 27 alloys in ${{\mathcal{D}}}_{\text{CALPHAD}}$ are used as the test set to evaluate the HEA recall rate. The model learned from the 216 alloys in the training set is used to rank the other 2,709 alloys, including the 27 in the test set. This cross-validation is also repeated 100 times to ensure the reliable evaluation of the HEA-recommendation performance.

By contrast, ${{\mathcal{D}}}_{\text{AFLOW}}$ and ${{\mathcal{D}}}_{\text{LTVC}}$ contain both binary and ternary alloys. Owing to the information obtained from both types of alloys, we can learn a similarity matrix between the various elements, elements and binary combinations thereof, and binary element combinations obtained from the training set sampled from ${{\mathcal{D}}}_{\text{AFLOW}}$ and ${{\mathcal{D}}}_{\text{LTVC}}$. We can build a model to rank all of the possible candidates for binary and ternary alloys other than those in the training set. There are 3,276 hypothetical binary and ternary alloys showing equivalent components that can be generated from the 27 elements in ${\mathcal{E}}$, 558 of which are contained in ${{\mathcal{D}}}_{\text{AFLOW}}$. As no information is available for the other 2,718 alloys, they are ranked by the constructed model. We apply ninefold cross-validation to ${{\mathcal{D}}}_{\text{AFLOW}}$ and use 496 of the 558 alloys in ${{\mathcal{D}}}_{\text{AFLOW}}$ as the training set. The remaining 62 alloys in ${{\mathcal{D}}}_{\text{AFLOW}}$ are used as the test set to evaluate the HEA recall rate. The model learned from the 496 alloys in the training set is used to rank the other 2,780 alloys including the 62 in the test set. The same evaluation method is applied to ${{\mathcal{D}}}_{\text{AFLOW}}$.

A similar experiment is conducted with ${{\mathcal{D}}}_{\text{LTVC}}$ to evaluate the HEA-recommendation performance of the proposed ERS. Note that although${{\mathcal{D}}}_{\text{LTVC}}$ contains the same alloys as ${{\mathcal{D}}}_{\text{AFLOW}}$, the target properties of the alloys are dissimilar as the values are estimated using different computation methods^42,43^.

It should be noted that owing to the computational cost, these experiments do not use the selected alloys (that is, those in the test set) to improve the accuracy of the HEA-recommendation model for the next trial. A recommendation model based on the results of previous trials may work more accurately.

### Experimental settings for evaluation of extrapolation capability

As ${{\mathcal{D}}}_{\text{AFLOW}}$ contains both binary and ternary alloys, we can learn the similarities between the various elements and binary combinations thereof. Consequently, we can apply the ERS to ${{\mathcal{D}}}_{\text{AFLOW}}$ to rank the 17,550 quaternary alloys comprising the 27 elements contained in ${\mathcal{E}}$. Furthermore, ${{\mathcal{D}}}_{\text{AFLOW}}$ and ${{\mathcal{D}}}_{\,\text{AFLOW}}^{\text{quaternary}\,}$ are both used to build a recommender system that ranks all of the possible candidates (that is, 80,730 alloys) for synthesizing quinary HEAs. The 754 quaternary HEAs in ${{\mathcal{D}}}_{\,\text{AFLOW}}^{\text{quaternary}\,}$ and 129 quinary HEAs in ${{\mathcal{D}}}_{\,\text{AFLOW}}^{\text{quinary}\,}$ are used to monitor the HEA recall rate for recommending quaternary and quinary HEAs, respectively. Moreover, similar experiments are conducted on ${{\mathcal{D}}}_{\text{LTVC}}$, ${{\mathcal{D}}}_{\,\text{LTVC}}^{\text{quaternary}\,}$ and ${{\mathcal{D}}}_{\,\text{LTVC}}^{\text{quinary}\,}$ to evaluate the HEA-recommendation performance of the ERS.

### Synthesis of FeMnCoNi HEA thin film

As a case study, we fabricated a HEA film of Fe_0.25_Co_0.25_Mn_0.25_Ni_0.25_. A 100-nm-thick thermal oxidized SiO_2_/Si(100) substrate was used. After the organic solvent and deionized water cleaning, the substrate was loaded in a combinatorial multitarget radio frequency sputtering system (COMET, CMS-6400). To identify the stable crystal structure and its composition dependence, a composition spread film was fabricated by the combinatorial method^44^. For the composition spread film, we used two targets of FeCoMn (1:1:1) and nickel (3N grade). The base pressure was below 1 × 10^−5^ Pa and argon gas pressure was set as 0.3 Pa. To adjust the deposition rate as 0.23 ± 0.01 nm s^–1^, radio frequency sputtering powers of FeCoMn and nickel targets were set at 100 W and 120 W, respectively. To enhance the crystallinity, the sample was annealed at 400 °C for 30 min under a vacuum condition below 6 × 10^−3^ Pa (Advanced RIKO, MILA-3000).

Figure 5b shows the sample structure. The composition film layer consists of three layers. One is a single FeCoMn layer with a thickness of 0.25 nm. The other layers are composition spread film formed by FeCoMn and nickel layers. For the composition-spread film deposition, during the FeCoMn layer deposition, a mask moved 18.5 mm at constant speed from a point 1.5 mm from the edge of the substrate to another end where the film thickness gradually changed. After that, the targets were changed to nickel. The mask moved to the opposite direction during the nickel film deposition. The total thickness of one unit of the 1-*x*(FeCoMn)–*x*Ni composition spread layer/FeCoMn stack structure is 0.5 nm. Alternating between the three deposition steps created composition-spread region with a width of 18.5 mm. The total film thickness in the composition-spread region was set to 100 nm. The composition spread was confirmed by an X-ray fluorescence spectrometer (Shimadzu, μEDX-1400) with a measuring spot diameter of 50 μm, as shown in Supplementary Fig. 5.

The crystal structure was identified by XRD. An XRD system with a 5 kW rotating anode copper target X-ray source and a high-resolution two-dimensional detector (BRUKER AXS, D8 Discover Super Speed with GADDS) was used to determine the crystal structure. The two-dimensional detector system can detect part of the Debye–Scherrer ring rapidly and two-dimensionally^45^

In the evaluation of the phase separation temperature and magnetization properties of the other FeCoMn-X compositions, we found that the phase separation and inflection point were observed near 400 °C. We thus set the annealing temperature as 400 °C. In the reported experiment, the annealing was performed at only 400 °C; however, for FeCoMnNi, structural changes at higher temperatures are expected and are currently under investigation. Supplementary Fig. 7 shows the XRD patterns of the sample as deposited and annealed. The BCC phase was confirmed for the annealed thin film sample at the equiatomical composition of FeCoMnNi (*x* = 0.25). Even at room temperature, a weak peak of the BCC can be observed for the FeCoM-rich composition.

### Supplementary information

Supplementary Information Supplementary Figs. 1–7, Discussion and Tables 1–4.

### Source data

Source Data Fig. 2 Statistical Source Data.

Source Data Fig. 3 Statistical Source Data.

Source Data Fig. 4 Statistical Source Data.

Source Data Fig. 5 Statistical Source Data and unprocessed figures.

## Data Availability

Datasets related to this article are deposited to the Zenodo repository^46^. Source Data are provided with this paper.

## Code availability

The full code, along with basic examples (1) showing the usage of commender system, (2) illustrating the similarity measurement and (3) explaining the method to evaluate the recommender system using an experiment with *k*-folds cross-validation, have been deposited to Code Ocean repository^47^.
