# Supplementary material for: Evidence-based recommender system for high-entropy alloys
Source: Nat Comput Sci. 2021 Jul 19;1(7):470–8. doi: 10.1038/s43588-021-00097-w (PMC10766533; doi:10.1038/s43588-021-00097-w)
Supplement: Supplementary file 1 — Supplementary Figs. 1–7, Discussion and Tables 1–4. [file 43588_2021_97_MOESM1_ESM.pdf]

nature portfolio

https://doi.org/10.1038/s43588-021-00097-w

Supplementary information

Evidence-based recommender system for high-entropy alloys

In the format provided by the authors and unedited

Supplementary Information

Minh-Quyet Ha,[1] Duong-Nguyen Nguyen,[1] Viet-Cuong Nguyen,[2] Takahiro Nagata,[3] Toyohiro Chikyow,[4] Hiori Kino,[4] Takashi Miyake,[5] Thierry Denœux,[6] Van-Nam Huynh,[1] and Hieu-Chi Dam[1]

[1)] *Japan Advanced Institute of Science and Technology, 1-1 Asahidai, Nomi, Ishikawa 923-1292, Japan*

[2)] *HPC SYSTEMS Inc., Minato, Tokyo 108-0022, Japan*

[3)] *RCFM, National Institute for Materials Science, 1-2-1 Sengen, Tsukuba, Ibaraki 305-0044, Japan*

[4)] *MaDIS, National Institute for Materials Science, 1-2-1 Sengen, Tsukuba, Ibaraki 305-0044, Japan*

[5)] *CD-FMat, AIST, 1-1-1 Umezono, Tsukuba, Ibaraki 305-8568, Japan*

[6)] *Université de technologie de Compiègne, CNRS, UMR 7253 Heudiasyc, Compiègne, France*

(*Electronic mail: dam@jaist.ac.jp)

(Dated: 5 June 2021)

I. ILLUSTRATIVE EXAMPLES

The following examples provide explanations of how the evidence theory work to learn the similarity and infer the HEA formation for new element combinations, identifying equiatomic alloys.

Example 1: Suppose we have collected four pairs of alloys from experiments. Three of those pairs are alloys that both form HEA phase: $pair_1 = (\{A^1, B^1, C^1, D\}, \{A^1, B^1, C^1, E\})$; $pair_2 = (\{A^2, B^2, C^2, D\}, \{A^2, B^2, C^2, E\})$; and $pair_3 = (\{A^3, B^3, C^3, D\}, \{A^3, B^3, C^3, E\})$. The fourth pair $pair_4 = (\{A^4, B^4, C^4, D\}, \{A^4, B^4, C^4, E\})$ is different from the other three, in which $\{A^4, B^4, C^4, D\}$ forms HEA phase while $\{A^4, B^4, C^4, E\}$ does not form HEA phase. We consider each pair as a source of evidence support that $\{D\}$ is similar to $\{E\}$ in term of substitutability to form the HEA phase. Each evidence is modeled using mass function as follows:

$$
\begin{aligned}
&m_{pair_1}^{\{C\},\{D\}}(\{similar\}) = 0.1,\\
&m_{pair_1}^{\{C\},\{D\}}(\{dissimilar\}) = 0,\\
&m_{pair_1}^{\{C\},\{D\}}(\{similar, dissimilar\}) = 0.9
\end{aligned}
$$

$$
\begin{aligned}
&m_{pair_2}^{\{C\},\{D\}}(\{similar\}) = 0.1,\\
&m_{pair_2}^{\{C\},\{D\}}(\{dissimilar\}) = 0,\\
&m_{pair_2}^{\{C\},\{D\}}(\{similar, dissimilar\}) = 0.9
\end{aligned}
$$

$$
\begin{aligned}
&m_{pair_3}^{\{C\},\{D\}}(\{similar\}) = 0.1,\\
&m_{pair_3}^{\{C\},\{D\}}(\{dissimilar\}) = 0,\\
&m_{pair_3}^{\{C\},\{D\}}(\{similar, dissimilar\}) = 0.9
\end{aligned}
$$

$$
\begin{aligned}
&m_{pair_4}^{\{C\},\{D\}}(\{similar\}) = 0,\\
&m_{pair_4}^{\{C\},\{D\}}(\{dissimilar\}) = 0.1,\\
&m_{pair_4}^{\{C\},\{D\}}(\{similar, dissimilar\}) = 0.9
\end{aligned}
$$

The three pieces of evidence are combined using the Dempster' rule of combination to accumulate the believe that $\{D\}$ is similar to $\{E\}$:

$$m^{\{C\},\{D\}}(\{similar\}) = 0.25,$$
$$m^{\{C\},\{D\}}(\{dissimilar\}) = 0.075,$$
$$m^{\{C\},\{D\}}(\{similar, dissimilar\}) = 0.675$$

Next, if we observed (included in the data) that the HEA phase exists for alloy $\{G, H, I, D\}$, the ERS (which focuses on finding some chance for discovering new combination of elements that the HEA phase exist and ignores the belief regarding $\neg HEA$) will consider that there is some believe that the HEA phase also exists for $\{G, H, I, E\}$ (by substituting $\{D\}$ with $\{E\}$). The evidence is modeled using mass function as follows:

$$m^{\{G,H,I,E\}}_{\{G,H,I,D\},\{D\}\leftarrow\{E\}}(\{\neg HEA\}) = 0,$$
$$m^{\{G,H,I,E\}}_{\{G,H,I,D\},\{D\}\leftarrow\{E\}}(\{HEA\}) = m^{C,D}(\{similar\}) = 0.25,$$
$$m^{\{G,H,I,E\}}_{\{G,H,I,D\},\{D\}\leftarrow\{E\}}(\{HEA, \neg HEA\}) = 1 - m^{C,D}(\{similar\}) = 0.75$$

Example 2: In a same manner but for an extrapolative recommendation: if the HEA phases exist for all the alloys in the three following pairs: $pair_1 = (\{A^1, B^1, C\}, \{A^1, B^1, D, E\})$, $pair_2 = (\{A^2, B^2, C\}, \{A^2, B^2, D, E\})$, $pair_3 = (\{A^3, B^3, C\}, \{A^3, B^3, D, E\})$. In the fourth pair $pair_4 = (\{A^4, B^4, C\}, \{A^4, B^4, D, E\})$, $\{A^4, B^4, C\}$ forms HEA phase while $\{A^4, B^4, D, E\}$ does not form HEA phase. The algorithm will accumulate the believe that $\{C\}$ is similar to $\{D, E\}$ as follows:

$$m^{\{C\},\{D,E\}}(\{similar\}) = 0.25,$$
$$m^{\{C\},\{D,E\}}(\{dissimilar\}) = 0.075,$$
$$m^{\{C\},\{D,E\}}(\{similar, dissimilar\}) = 0.675$$

Consequently, if we observed (included in the data) that the HEA phase exists for $\{G, H, I, C\}$, the algorithm (which focuses on finding some chance for discovering new combination of elements that the HEA phase exist and ignores the belief regarding $\neg HEA$) will consider that there is some believe that the HEA phase also exists for $\{G, H, I, D, E\}$ (by substituting $\{C\}$ with $\{D, E\}$).

$$m^{\{G,H,I,D,E\}}_{\{G,H,I,C\},\{C\}\leftarrow\{D,E\}}(\{\neg HEA\}) = 0,$$
$$m^{\{G,H,I,D,E\}}_{\{G,H,I,C\},\{C\}\leftarrow\{D,E\}}(\{HEA\}) = m^{C,D}(\{similar\}) = 0.25,$$
$$m^{\{G,H,I,D,E\}}_{\{G,H,I,C\},\{C\}\leftarrow\{D,E\}}(\{HEA, \neg HEA\}) = 1 - m^{C,D}(\{similar\}) = 0.75$$

II. ALLOYS DATA SETS

In the evaluation experiments, we use eight data sets consisting of binary, ternary, quaternary, and quinary alloys comprising multiple equiatomically combined elements. The data sets consist of data from experiments and calculations. In this section, we will follow Ref. 35 to describe the data sets. The alloys contained in the data sets comprise $\mathcal{E} = \{$ Fe, Co, Ir, Cu, Ni, Pt, Pd, Rh, Au, Ag, Ru, Os, Si, As, Al, Tc, Re, Mn, Ta, Ti, W, Mo, Cr, V, Hf, Nb, and Zr$\}$. Supplementary Figure 2 shows the proportion of 27 elements in the data sets. Any alloy contained in the following data sets is considered as an HEA if its order-disorder transition temperature is below its melting temperature.

- $\mathcal{D}_{\mathrm{ASMI16}}$: The order-disorder transition temperatures (T_c^{exp}) and melting temperatures (T_m^{exp}) of the alloys are both experimentally evaluated[1]. All of the alloys contained in $\mathcal{D}_{\mathrm{ASMI16}}$ show an order-disorder transition temperature below their melting temperature ($T_c^{\mathrm{exp}} < T_m^{\mathrm{exp}}$).

- $\mathcal{D}_{\mathrm{CALPHAD}}$: The order-disorder transition temperatures ($T_c^{\star}$) and melting temperatures ($T_m^{\star}$) of the alloys are both predicted using calculated-phase-diagram (CALPHAD) calculations[2–4] based on the temperatures for some binary alloys (three possible for each ternary alloy) found in the Thermo-Calc software SSOL5 database[5]. Similar to the $\mathcal{D}_{\mathrm{ASMI16}}$ data set, the $\mathcal{D}_{\mathrm{CALPHAD}}$ data set only contains the alloys satisfying $T_c^{\star} < T_m^{\star}$.

- $\mathcal{D}_{\mathrm{AFLOW}}$, $\mathcal{D}_{\mathrm{AFLOW}}^{\mathrm{quaternary}}$, and $\mathcal{D}_{\mathrm{AFLOW}}^{\mathrm{quinary}}$: The order-disorder transition temperatures (T_c^{AFLOW}) of the alloys contained in these data sets are estimated using the automatic flow (AFLOW) convex-hull database[6]. The melting temperatures T_m^{exp} and $T_m^{\star}$ are applied to the binary and ternary alloys, respectively. The alloy is considered as an HEA if $T_c^{\mathrm{AFLOW}} < T_m^{\mathrm{exp}}$ for binary alloys and $T_c^{\mathrm{AFLOW}} < T_m^{\star}$ for ternary, quaternary, and quinary alloys).

- $\mathcal{D}_{\mathrm{LTVC}}$, $\mathcal{D}_{\mathrm{LTVC}}^{\mathrm{quaternary}}$, and $\mathcal{D}_{\mathrm{LTVC}}^{\mathrm{quinary}}$: These data sets contain the same alloys as those contained in data sets $\mathcal{D}_{\mathrm{AFLOW}}$, $\mathcal{D}_{\mathrm{AFLOW}}^{\mathrm{quaternary}}$, and $\mathcal{D}_{\mathrm{AFLOW}}^{\mathrm{quinary}}$, respectively. However, the properties of the alloys contained in these data sets are predicted using the method of Lederer, Toher, Vecchio, and Curtarolo (LTVC)[7]. *Ab-initio* calculations are used

to estimate the order-disorder transition temperatures (T_c^{LTVC}) of the alloys contained in these data sets. In addition, the T_m values are the same as those of the alloys contained in the AFLOW data sets. Any alloy in these data sets is predicted as an HEA if $T_c^{\mathrm{LTVC}} < T_m^{\mathrm{exp}}$ for binary alloys and $T_c^{\mathrm{LTVC}} < T_m^{\star}$ for ternary, quaternary, and quinary alloys

Note that $\mathcal{D}_{\mathrm{ASMI16}}$ and $\mathcal{D}_{\mathrm{CALPHAD}}$ only contain confirmed and predicted HEAs, respectively. Therefore, although we assume that the properties of all the other binary or ternary alloys (not included in the data set) have not yet been confirmed, we do not assume that those alloys are not HEAs.

III. DIFFERENCES BETWEEN SIMILARITY MATRICES LEARNED FROM $\mathcal{D}_{\mathrm{CALPHAD}}$ AND $\mathcal{D}_{\mathrm{AFLOW}}$

There are some notable differences between these results obtained from experiments with $\mathcal{D}_{\mathrm{CALPHAD}}$ and $\mathcal{D}_{\mathrm{AFLOW}}$. The similarity matrix learned from $\mathcal{D}_{\mathrm{AFLOW}}$ shows that Au and Ag are very similar (Supplementary Figure 3 b). Furthermore, both are similar to V, Mn, and Al but not to other late transition metals (Supplementary Figure 3 a). Mn is also similar to Tc, Re, and Cr but not to the other early transition metals. However, Tc and Re are somewhat similar to the other early transition metals. Furthermore, Zr is somewhat similar to the late transition metals, but different from the early transition metals. Clearly, these results are different from that obtained from $\mathcal{D}_{\mathrm{CALPHAD}}$ owing to the difference between the predicted label (HEA or $\neg HEA$) for the Zr-containing alloys recommended based on CALPHAD and AFLOW calculations, as listed in Supplementary Table 1. Al, Si, and As are all similar to each other and to Fe and Co (Supplementary Figure 3 a). However, Al is similar to V, Cr, and Mn but not to Ti, whereas Si and As are very similar to Ti but not to V or Cr.

IV. MONITORING HEA RECALL RATIOS IN TEST SET

A. Evaluation of HEA-recommendation capability by cross-validation

In the experiment with $\mathcal{D}_{\mathrm{ASMI16}}$, the result shows that the ERS can significantly reduce the number of trials required to recall all the HEAs in the test set compared to the competitor systems (Supplementary Figure 4 a). The proposed ERS requires less than 12, 25, and 80% of all the possible trials to recall one-half, three-quarters, and all the HEAs in the test set, respectively (Supplementary Table 2). In the $\mathcal{D}_{\mathrm{CALPHAD}}$ experiment, the ERS requires less than 2 and 5% of all the possible trials to recall one-half and three-quarters of the HEAs in the test set, respectively, which are the fewest trials required among all the recommender systems (Supplementary Figure 4 b and Supplementary Table 2). Interestingly, in the $\mathcal{D}_{\mathrm{ASMI16}}$ and $\mathcal{D}_{\mathrm{CALPHAD}}$ experiments, the supervised-method-based recommender systems either approximately randomly selected possible HEAs (Naïve Bayes and decision tree) or could not rank any (logistic regression and SVM) at all because these data sets contain only positively labeled HEAs.

The result in $\mathcal{D}_{\mathrm{AFLOW}}$ experiment demonstrates that the ERS also outperforms the competitor systems in recalling one-half of the HEAs in the test set. However, the ERS cannot reliably recall the one-quarter of the HEAs remaining in the test set because not enough evidence is available in the training data to make inferences about the remaining HEAs (Supplementary Figure 4 c and Supplementary Table 2). The $\mathcal{D}_{\mathrm{LTVC}}$ and $\mathcal{D}_{\mathrm{AFLOW}}$ experimental results are identical(Supplementary Figure 4 d). Although the ERS performs better than the other recommendation systems in recovering one-half of the test HEAs in the $\mathcal{D}_{\mathrm{LTVC}}$ data set (requiring only less than 3% of the number of possible trials), it cannot reliably recover the remaining one-quarter of the test HEAs owing to the lack of evidence in the training data (Supplementary Table 2).

B. Evaluation of HEA-recommendation capability by extrapolation

In the $\mathcal{D}^{\mathrm{quaternary}}_{\mathrm{AFLOW}}$ experiment, the ERS performs significantly better than the NMF-based recommender system, requiring less than 5 and 19% of the total number of possible HEA candidates to recall 50 and 75% of the HEAs in the test set, respectively (Supplementary Table 3). In the $\mathcal{D}^{\mathrm{quaternary}}_{\mathrm{LTVC}}$ experiment, the ERS and competitor matrix-based system devel-

oped using the first type of matrix representation require 13 and 32% and 14 and 41% of the total number of possible HEA candidates to recall 50 and 75% of the HEAs in the test set, respectively (Supplementary Table 3). Further investigation indicates that the ERS hardly recommends any quaternary alloys in $\mathcal{D}_{\text{LTVC}}^{\text{quaternary}}$ because these alloys cannot be generated by substituting elements in any of the ternary alloys in $\mathcal{D}_{\text{LTVC}}$ (Supplementary Table 4). Therefore, the properties of these alloys cannot be inferred from the evidence collected from $\mathcal{D}_{\text{LTVC}}$. As a result, the rankings obtained for these alloys are significantly low; therefore, the HEA recall rate is even lower than those obtained for randomly recommended HEAs. The results obtained for $\mathcal{D}_{\text{LTVC}}^{\text{quinary}}$ and $\mathcal{D}_{\text{AFLOW}}^{\text{quinary}}$ both show that the ERS drastically outperforms the capability of the competitor systems for recommending quinary HEAs. To recall 50, 75, and 100% of the HEAs from these data sets, 10–100 times fewer trials are required using the ERS than are required using the matrix-based recommender systems (Supplementary Table 3).

Supplementary Table 1. Comparison of the properties of alloys containing Zr in the two datasets $\mathcal{D}_{\mathrm{ASMI16}}$ and $\mathcal{D}_{\mathrm{CALPHAD}}$ to those predicted in $\mathcal{D}_{\mathrm{LTVC}}$ and $\mathcal{D}_{\mathrm{AFLOW}}$.

	$\mathcal{D}_{\mathrm{ASMI16}}$ 4 alloys	$\mathcal{D}_{\mathrm{CALPHAD}}$ 19 alloys
#in agreement with $\mathcal{D}_{\mathrm{AFLOW}}$	4	10
#disagreement with $\mathcal{D}_{\mathrm{AFLOW}}$	0	9
#in agreement with $\mathcal{D}_{\mathrm{LTVC}}$	3	10
#disagreement with $\mathcal{D}_{\mathrm{LTVC}}$	1	9

Supplementary Table 2. Ratio of number of trials (out of total number of possible trials) required to recall 50, 75, and 100% of HEAs in test set.

Data set	Model	Recall rates Half	Three-quarters	Full
$\mathcal{D}_{\text{ASMI16}}$	ERS	**12%**	**25%**	**80%**
	NMF (type 1)	16%	25%	92%
	NMF (type 2)	13%	26%	98%
	SVD (type 1)	31%	68%	99%
	SVD (type 2)	23%	64%	99%
	Decision Tree	77%	90%	99%
	Naïve Bayes	77%	90%	99%
	Logistic Regression	-	-	-
	SVM	-	-	-
$\mathcal{D}_{\text{CALPHAD}}$	ERS	**2%**	**5%**	92%
	NMF (type 1)	3%	7%	**89%**
	NMF (type 2)	3%	8%	93%
	SVD (type 1)	14%	28%	94%
	SVD (type 2)	17	37%	93%
	Decision Tree	39%	52%	94%
	Naïve Bayes	39%	52%	94%
	Logistic Regression	-	-	-
	SVM	-	-	-
$\mathcal{D}_{\text{AFLOW}}$	ERS	**2%**	8%	97%
	NMF (type 1)	3%	**6%**	96%
	NMF (type 2)	3%	6%	**85%**
	SVD (type 1)	16%	35%	99%
	SVD (type 2)	20%	50%	99%
	Decision Tree	31%	51%	99%
	Naïve Bayes	33%	53%	99%
	Logistic Regression	20%	29%	93%
	SVM	15%	26%	99%
$\mathcal{D}_{\text{LTVC}}$	ERS	**3%**	23%	97 %
	NMF (type 1)	4%	**6%**	96%
	NMF (type 2)	4%	7%	**86%**
	SVD (type 1)	14%	33%	99%
	SVD (type 2)	19%	52%	99%
	Decision Tree	32%	48%	99%
	Naïve Bayes	26%	42%	99%
	Logistic Regression	17%	26%	89%
	SVM	12%	26%	99%

Supplementary Table 3. Ratio of number of trials (out of total number of possible trials) required to recall 50, 75, and 100% of HEAs in test set by extrapolating HEA-recommendation capability.

Data set	Model	Recall rates: Half	Three-quarters	Full
$\mathcal{D}_{\text{AFLOW}}^{\text{quaternary}}$	ERS	**5%**	**19%**	**99%**
	NMF (type 1)	10%	24%	99%
	NMF (type 2)	50%	67%	99%
	SVD (type 1)	13%	32%	99%
	SVD (type 2)	53%	67%	99%
$\mathcal{D}_{\text{AFLOW}}^{\text{quinary}}$	ERS	**0.4%**	**1%**	**3%**
	NMF (type 1)	10%	56%	98%
	NMF (type 2)	9%	14%	47%
	SVD (type 1)	15%	27%	99%
	SVD (type 2)	8%	57%	99%
$\mathcal{D}_{\text{LTVC}}^{\text{quaternary}}$	ERS	**13%**	**32%**	**99%**
	NMF (type 1)	14%	41%	99%
	NMF (type 2)	50%	71%	99%
	SVD (type 1)	15%	39%	99%
	SVD (type 2)	53%	71%	99%
$\mathcal{D}_{\text{LTVC}}^{\text{quinary}}$	ERS	**0.07%**	**0.2%**	**2%**
	NMF (type 1)	11%	16%	47%
	NMF (type 2)	10%	53%	93%
	SVD (type 1)	15%	27%	99%
	SVD (type 2)	7%	54%	93%

Supplementary Table 4. List of 63 quaternary HEAs in $\mathcal{D}_{\mathrm{LTVC}}^{\mathrm{quaternary}}$ that no evidence about their properties is found.

FeAuRePd	AuNiPdOs	NiRePtOs	RhRePtOs	RePtOsAg
FeNiRePd	AuRhRePd	NiPdRuOs	RhPdOsAg	PdRuOsAg
FeMoOsAg	AuRhPdOs	NiPdCuOs	CoRePdRu	PdCuOsCr
FeRhPdOs	AuRePdRu	NiPdOsCr	CoRePdCu	ReCuPtOs
FeRePdRu	AuRePdCu	NiPdOsAg	CoRePdOs	RhRePdAg
FeRePdCu	AuRePdOs	MoRhPdOs	CoRePdAg	NiRePdAg
FeRePdOs	AuRePdAg	MoRePdOs	CoReRuPt	AuNiReAg
FeReRuAg	NiMoPdOs	MoRePtOs	CoRePtOs	ReRuPtOs
FeReOsAg	NiRhRePd	MoReOsAg	CoPdRuOs	RhRePdOs
FePdRuOs	NiRhPdOs	MoPdRuOs	CoRuPtOs	NiRePdOs
FePdCuOs	NiCoRePd	MoRuPtOs	RePdRuOs	FeCuOsAg
FePdOsCr	NiRePdRu	RhCoPdOs	RePdPtOs	
FeRuOsAg	NiRePdCu	RhRePdCu	RePdOsAg	

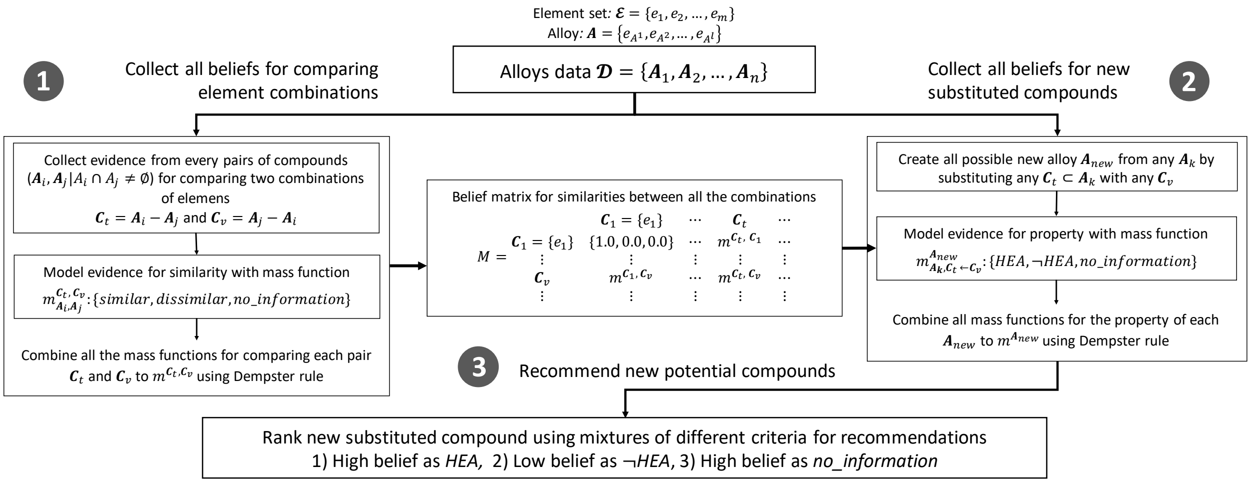

Supplementary Figure 1. Workflow chart illustrating three ERS stages required for recommending undiscovered HEAs. The data set $\mathcal{D}$ includes observed materials generated from a finite set $\mathcal{E}$ of elements.

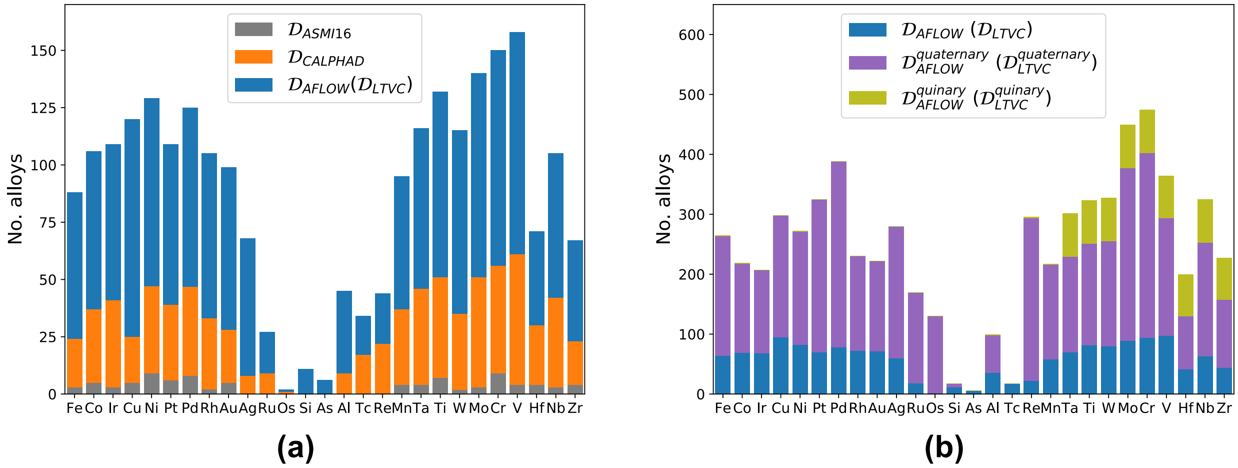

Supplementary Figure 2. Proportions of 27 elements in $\mathcal{D}_{\text{ASMI16}}$, $\mathcal{D}_{\text{CALPHAD}}$, $\mathcal{D}_{\text{AFLOW}}$, $\mathcal{D}_{\text{AFLOW}}^{\text{quaternary}}$, $\mathcal{D}_{\text{AFLOW}}^{\text{quinary}}$, $\mathcal{D}_{\text{LTVC}}$, $\mathcal{D}_{\text{LTVC}}^{\text{quaternary}}$, and $\mathcal{D}_{\text{LTVC}}^{\text{quinary}}$ data sets.

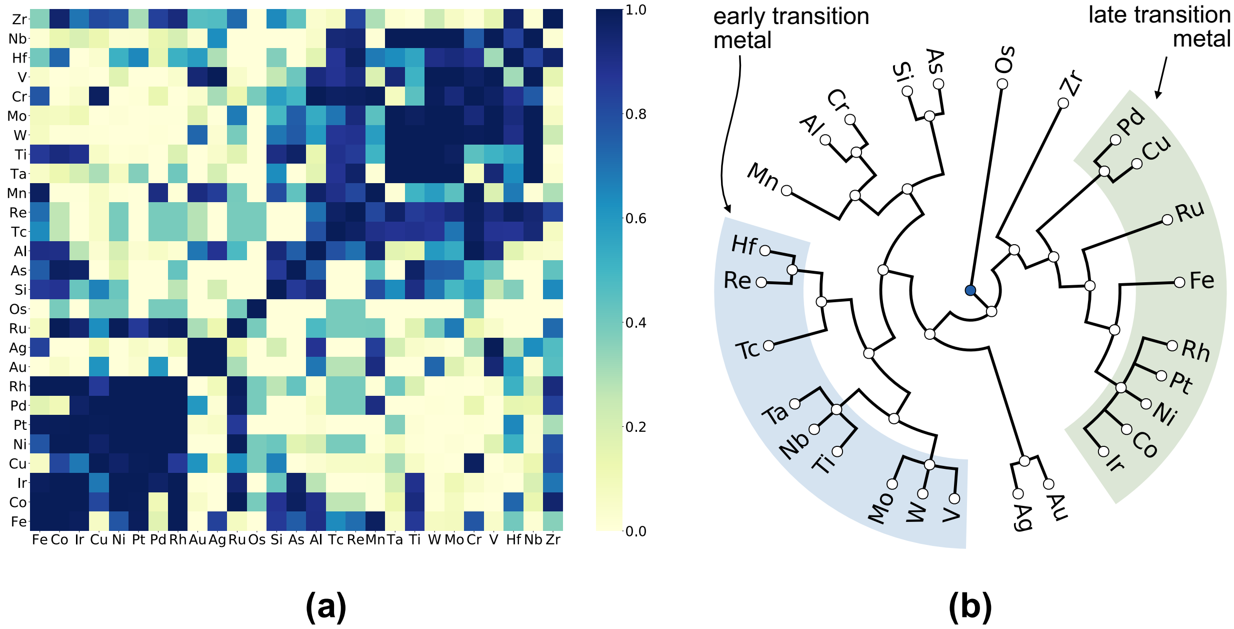

Supplementary Figure 3. (a) Heat maps for similarity matrices among 27 elements $\mathcal{E}$ obtained from$\mathcal{D}_{\mathrm{AFLOW}}$ data set. (b) Hierarchically clustered structure of all elements in $\mathcal{E}$ constructed using the similarity matrix and hierarchical agglomerative clustering. Blue and green regions indicate groups of early and late transition metals, respectively.

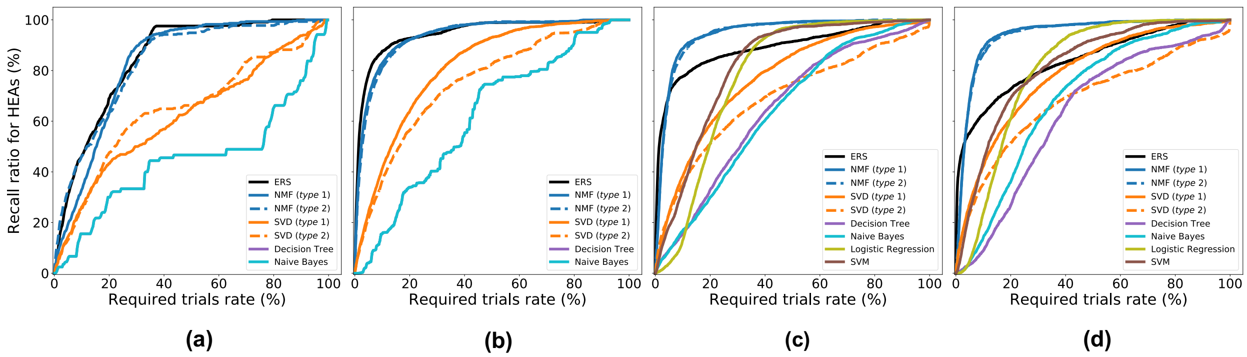

Supplementary Figure 4. Dependence of HEA recall ratio in the test sets on the number of trial required using k-fold cross-validation on (a) $\mathcal{D}_{\mathrm{ASMI16}}$, (b) $\mathcal{D}_{\mathrm{CALPHAD}}$, (c) $\mathcal{D}_{\mathrm{AFLOW}}$, and (d) $\mathcal{D}_{\mathrm{LTVC}}$ data sets.

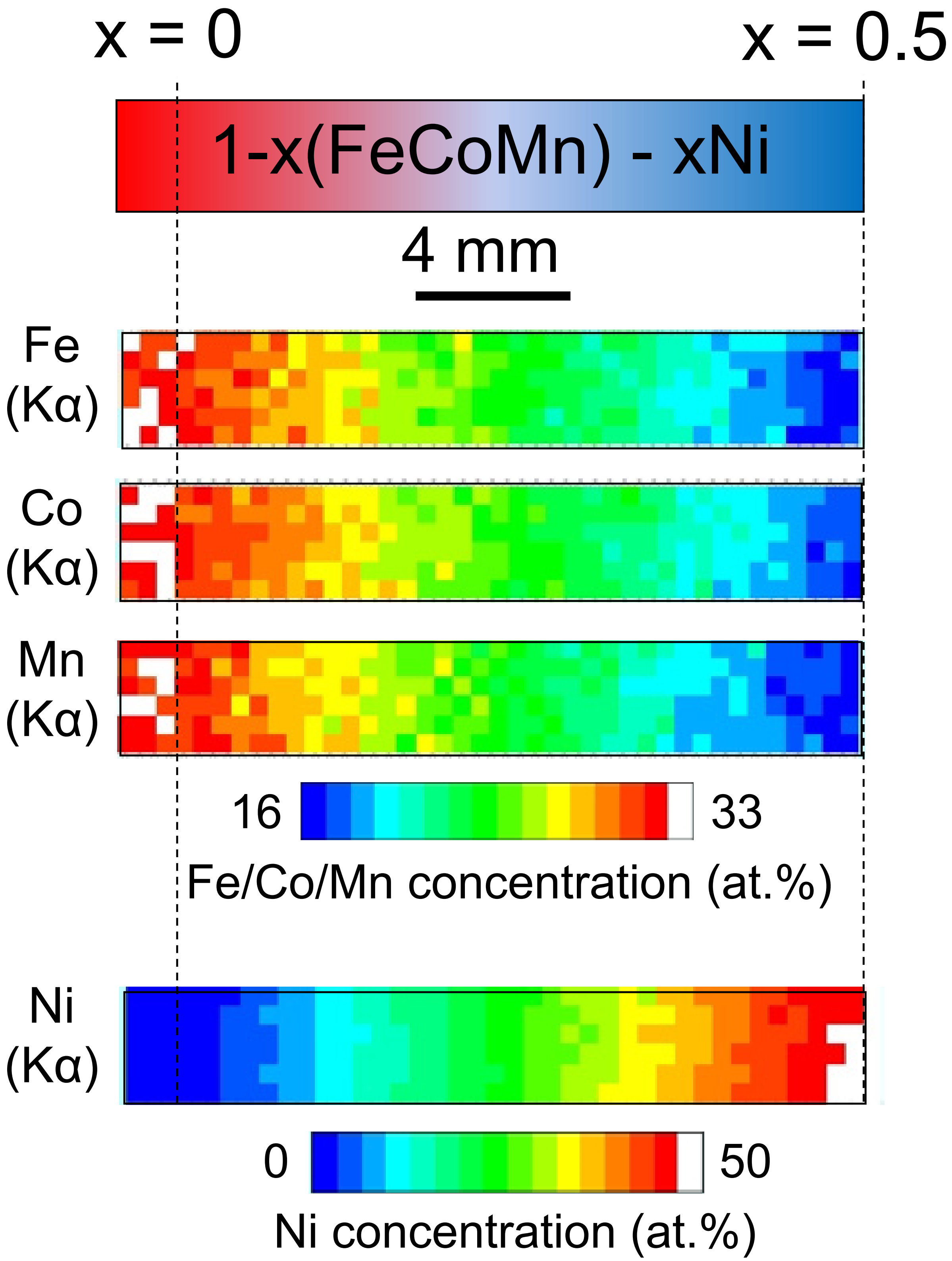

Supplementary Figure 5. Heatmapping image of Fe, Co, Mn, and Ni concentration estimated by EDX analysis. Composition was estimated from the XRF intensity of balk target materials and single-phase films of FeCoMn and Ni.

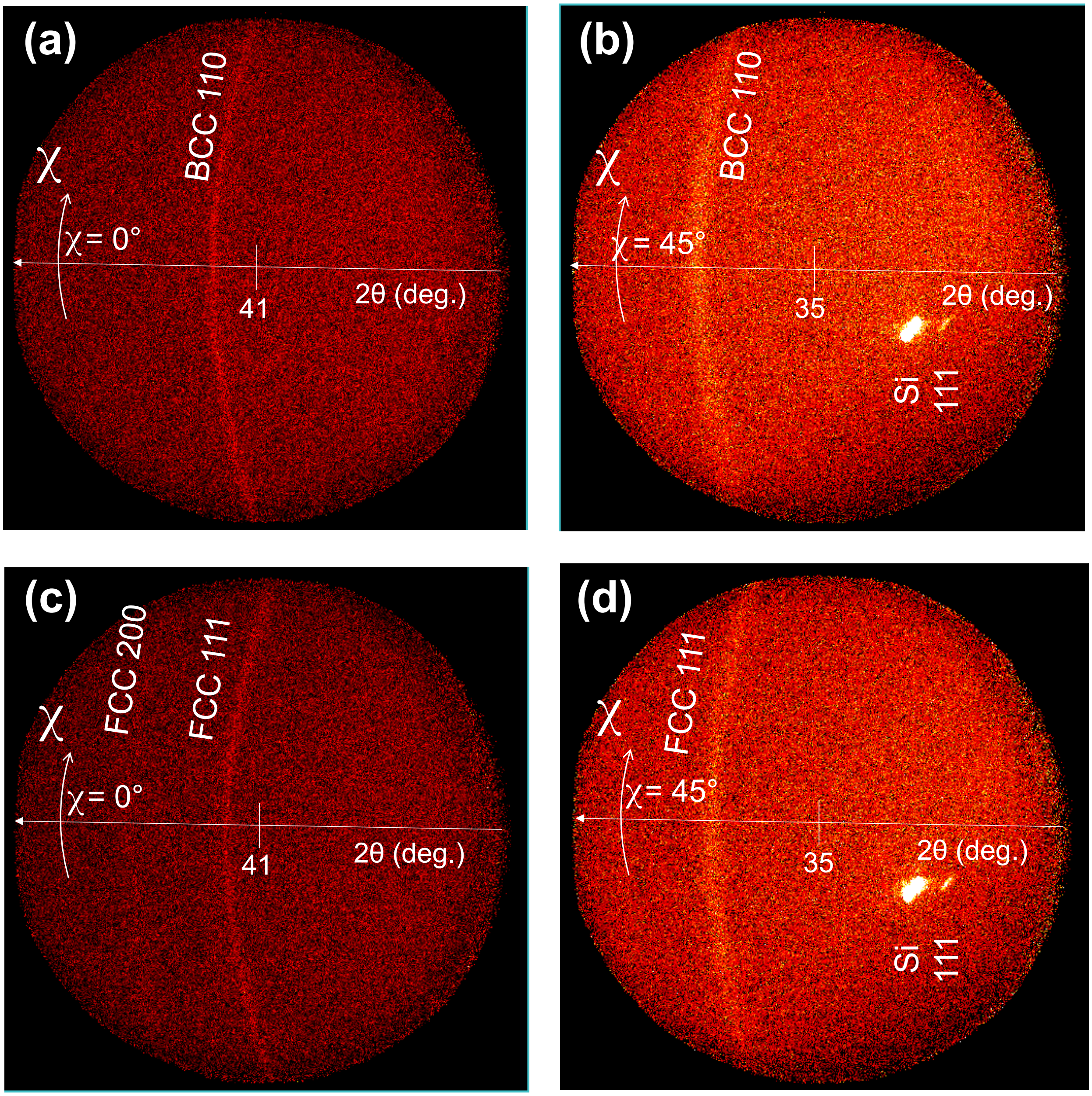

Supplementary Figure 6. 2D-XRD images at center γ angles of 0 and 45 $\circ$of FeCoMn-Ni films with low- (a,b) and high- (c,d) Ni concentrations. According to the powder diffraction pattern data base[8] (PDF 03-065-7519 and PDF 03-065-5131), for BCC, except for the reflection from (110), the signal intensities from other plane are not enough high to detect them in film form. So, the reflection from (110) is only detected. For FCC, in addition to the reflection from (111), the second strongest signal from (200) can barely be detected. The signals do not show no γ angle dependence, meaning the films are polycrystals in disordered crystal orientation.

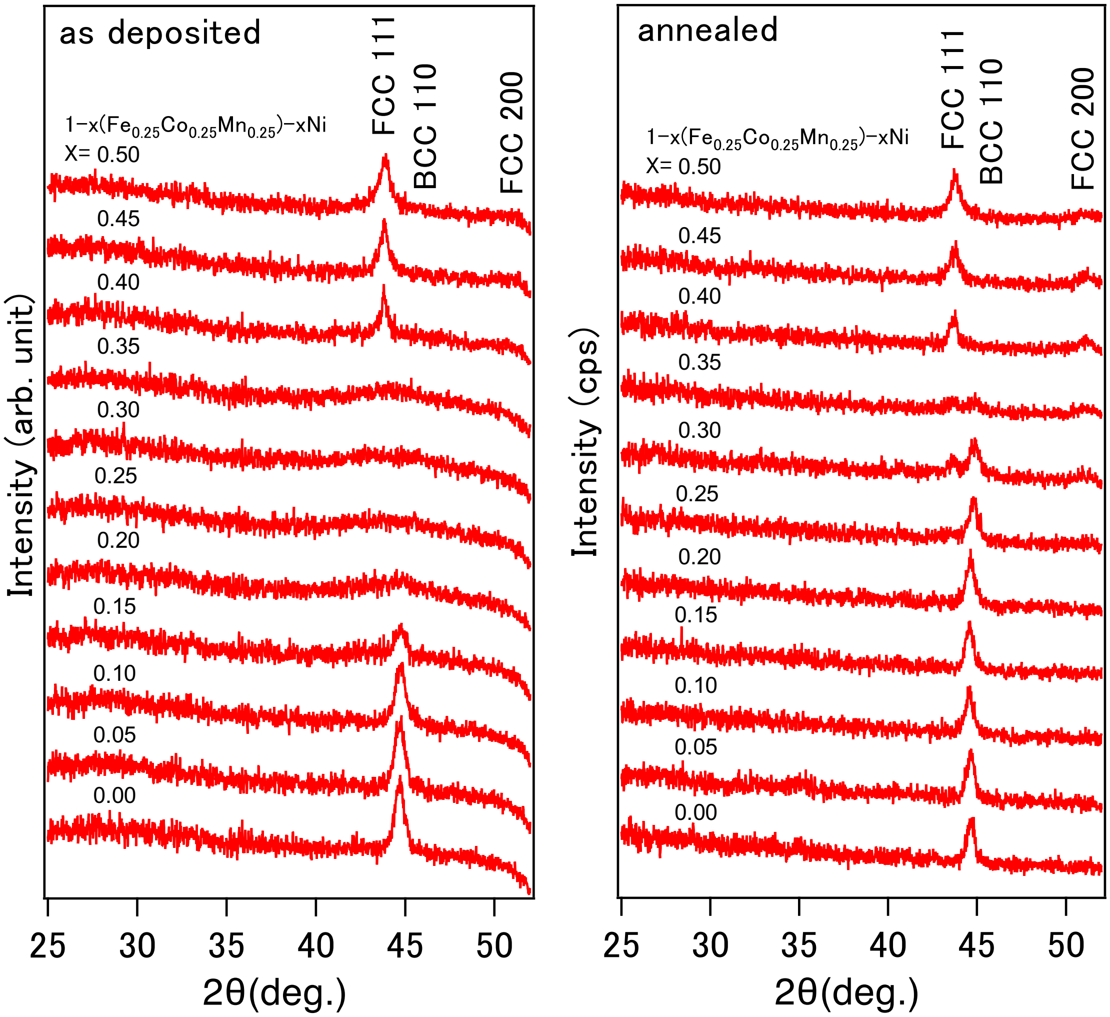

Supplementary Figure 7. XRD patterns of the as deposited and annealed at 400°C of FeCoMn-Ni film using an XRD system with a 5-kW rotating anode Cu target x-ray source. The BCC phase was confirmed for the annealed thin film sample at the equiatomical composition of FeCoMnNi (x=0.25)

REFERENCES

[1]"Binary Alloy Phase Diagrams," in *Alloy Phase Diagrams*, Vol. 3, edited by H. Okamoto, M. Schlesinger, and E. Mueller (ASM International, 2016).

[2]O. N. Senkov, J. D. Miller, D. B. Miracle, and C. Woodward, "Accelerated exploration of multi-principal element alloys with solid solution phases," Nat. Commun. **6**, 6529 (2015).

[3]D. Alman, "Searching for next single-phase high-entropy alloy compositions," Entropy **15**, 4504–4519 (2013).

[4]F. Zhang, C. Zhang, S. Chen, J. Zhu, W. Cao, and U. Kattner, "An understanding of high entropy alloys from phase diagram calculations," CALPHAD **45**, 1–10 (2014).

[5]J.-O. Andersson, T. Helander, L. Höglund, P. Shi, and B. Sundman, "Thermo-calc & dictra, computational tools for materials science," Calphad **26**, 273–312 (2002).

[6]C. Nyshadham, C. Oses, J. E. Hansen, I. Takeuchi, S. Curtarolo, and G. L. Hart, "A computational high-throughput search for new ternary superalloys," Acta Mater. **122**, 438–447 (2017).

[7]Y. Lederer, C. Toher, K. S. Vecchio, and S. Curtarolo, "The search for high entropy alloys: A high-throughput ab-initio approach," Acta Mater. **159**, 364–383 (2018).

[8]S. Gates-Rector and T. Blanton, "The powder diffraction file: a quality materials characterization database," Powder Diffr. **34**, 352–360 (2019).
