# Supplementary figures and images for: Evidence-based recommender system for high-entropy alloys
Source: Nat Comput Sci. 2021 Jul 19;1(7):470–8. doi: 10.1038/s43588-021-00097-w (PMC10766533; doi:10.1038/s43588-021-00097-w)

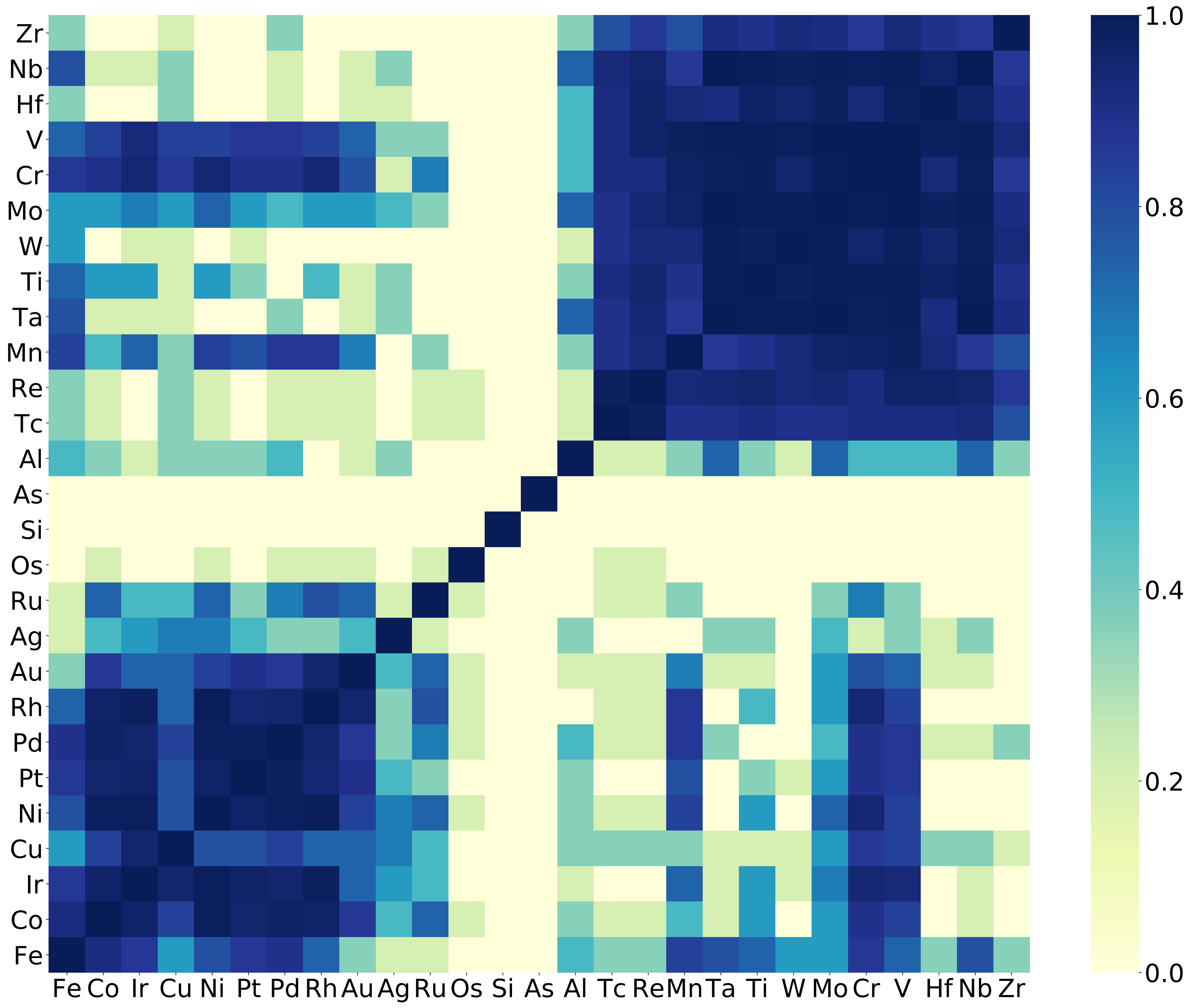

Supplement: Supplementary file 2 — Statistical Source Data. [file 43588_2021_97_MOESM2_ESM.zip › Figure_2/Figure_2b.pdf]

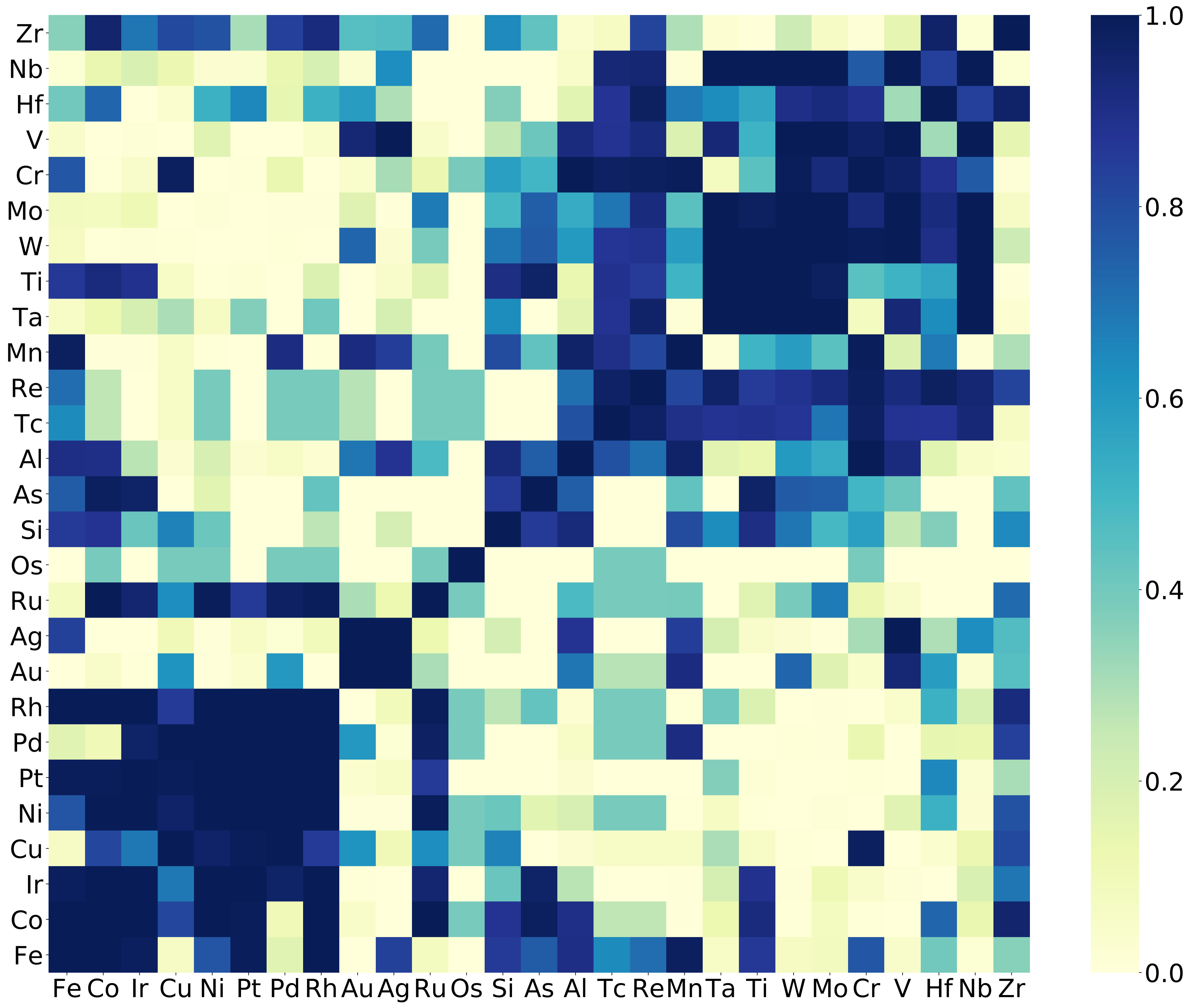

Supplement: Supplementary file 2 — Statistical Source Data. [file 43588_2021_97_MOESM2_ESM.zip › Figure_2/Figure_2c.pdf]

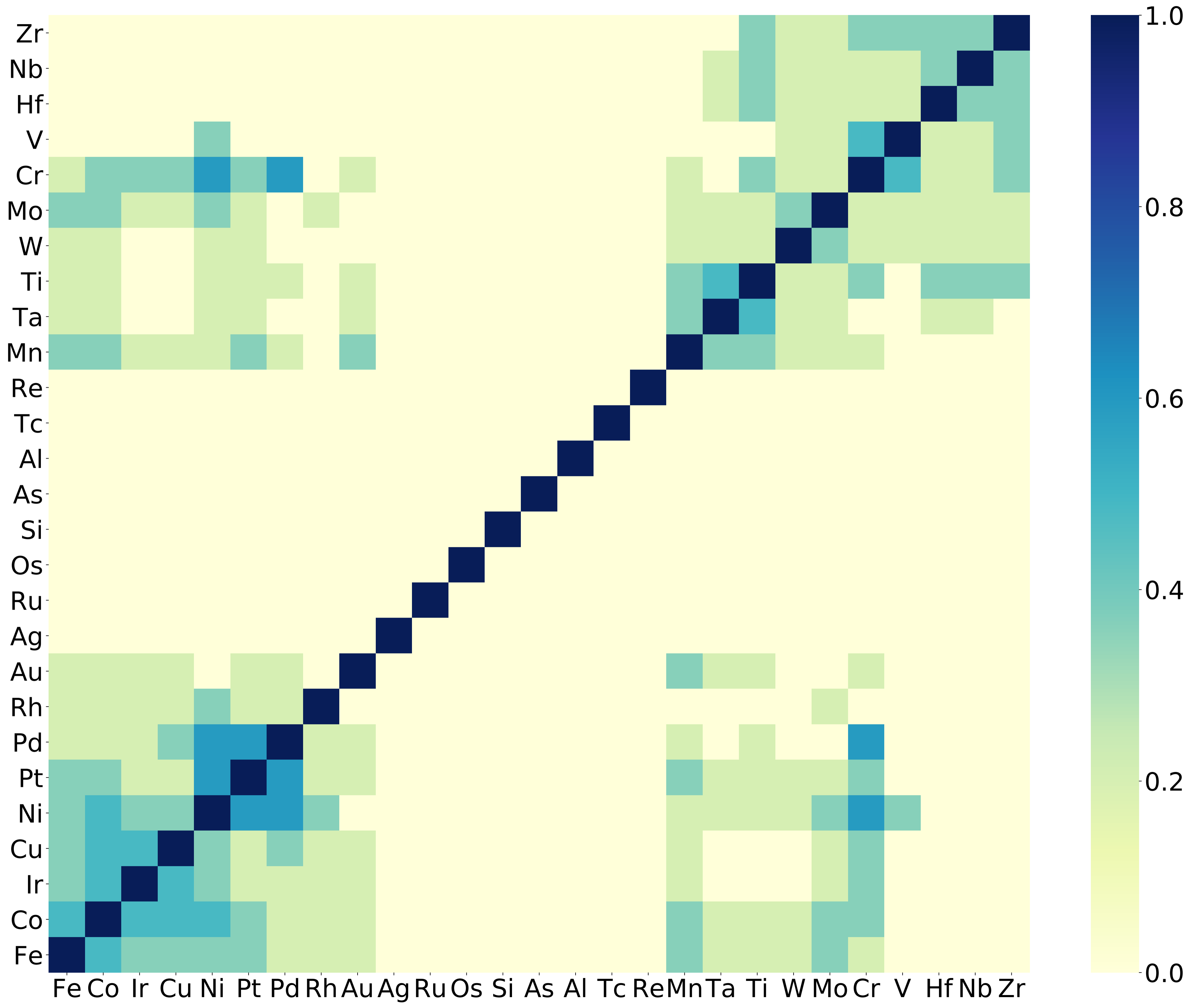

Supplement: Supplementary file 2 — Statistical Source Data. [file 43588_2021_97_MOESM2_ESM.zip › Figure_2/Figure_2a.pdf]

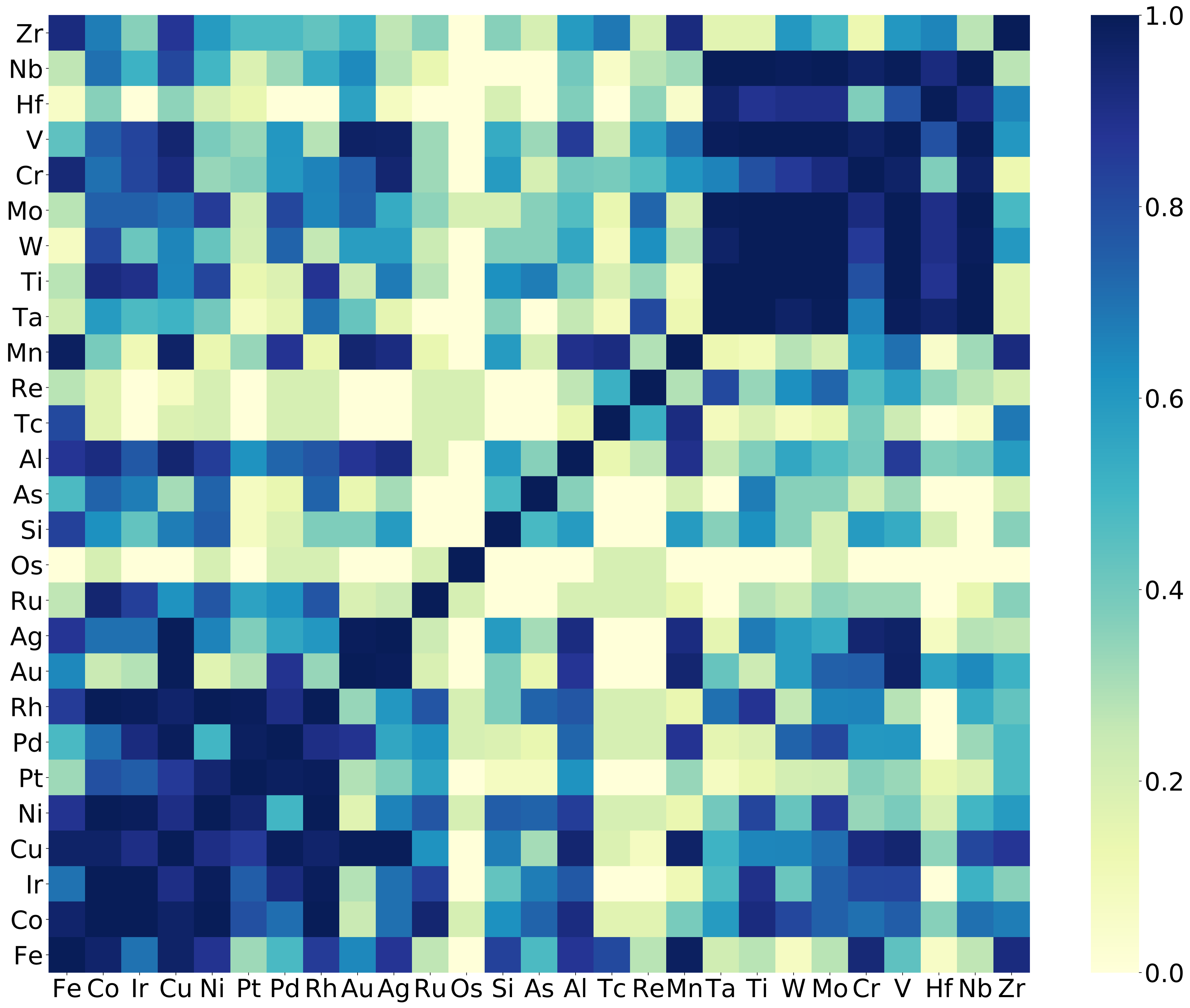

Supplement: Supplementary file 2 — Statistical Source Data. [file 43588_2021_97_MOESM2_ESM.zip › Figure_2/Figure_2d.pdf]

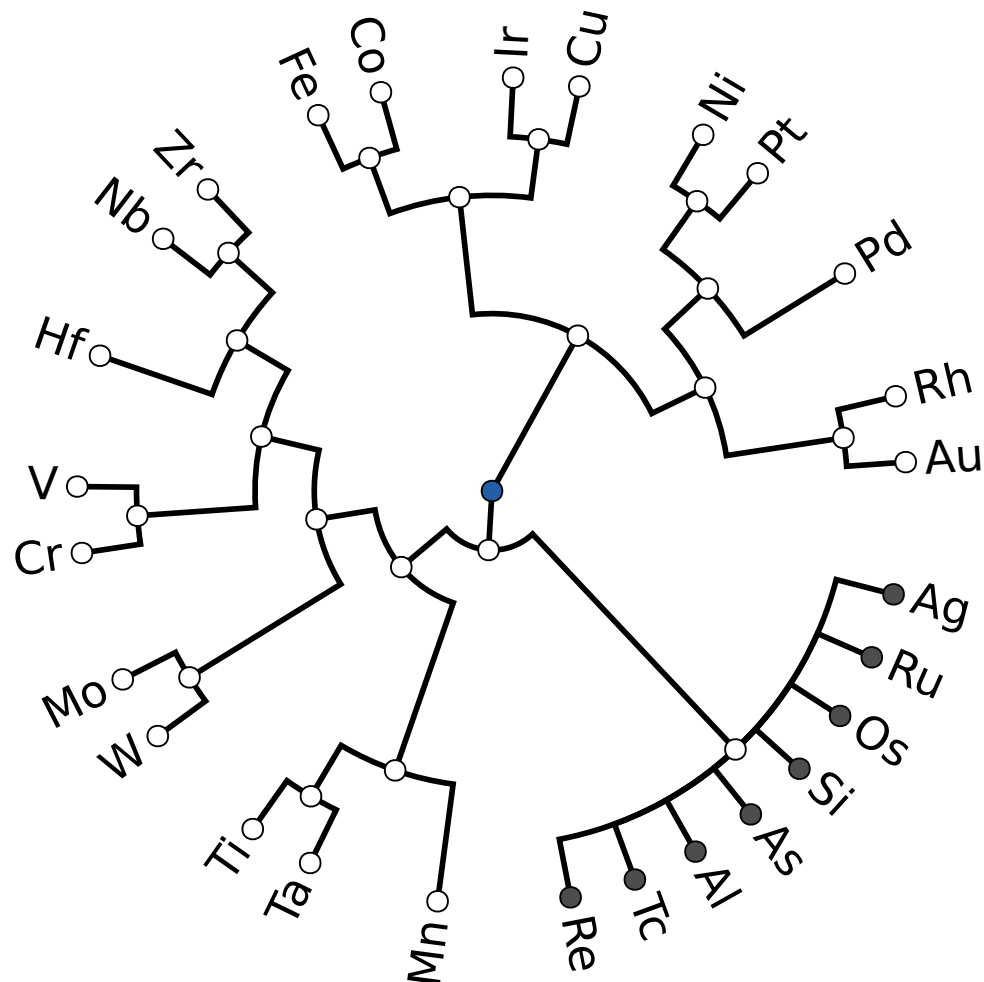

Supplement: Supplementary file 2 — Statistical Source Data. [file 43588_2021_97_MOESM2_ESM.zip › Figure_2/Figure_2e.pdf]

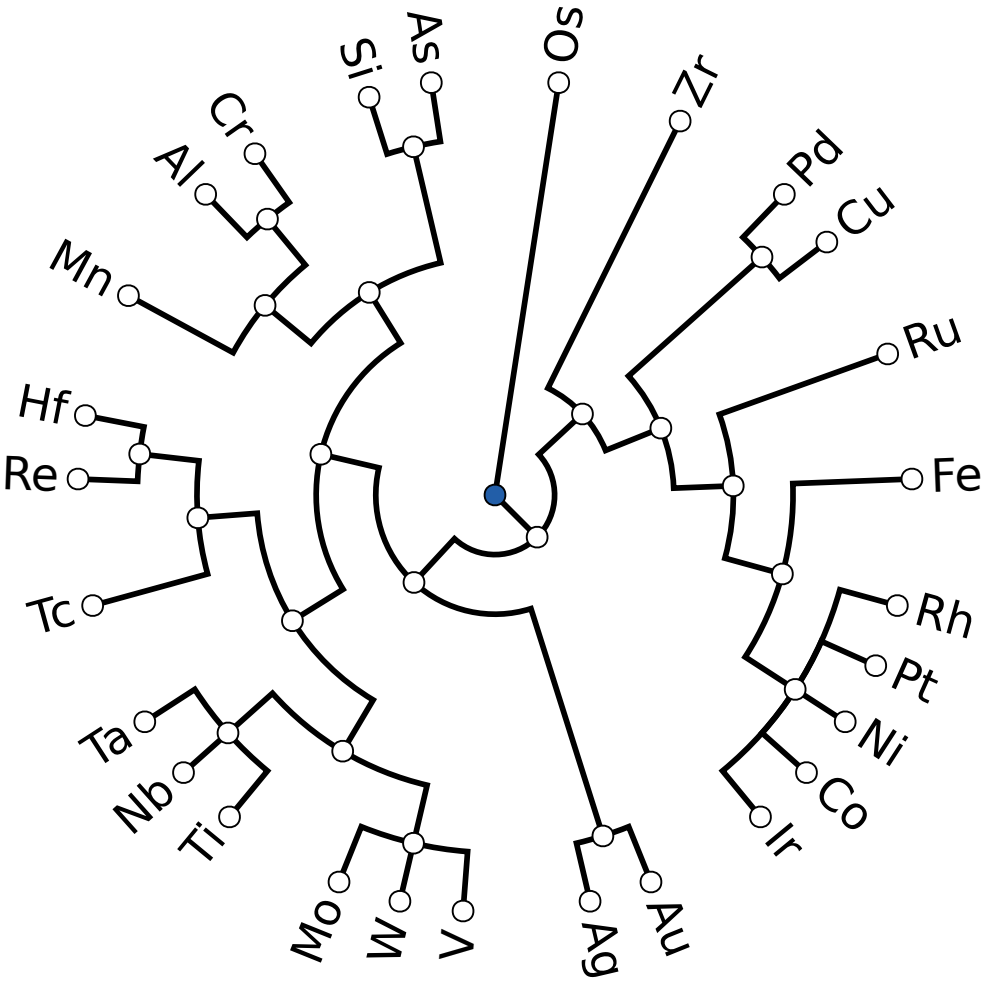

Supplement: Supplementary file 2 — Statistical Source Data. [file 43588_2021_97_MOESM2_ESM.zip › Figure_2/Figure_2g.pdf]

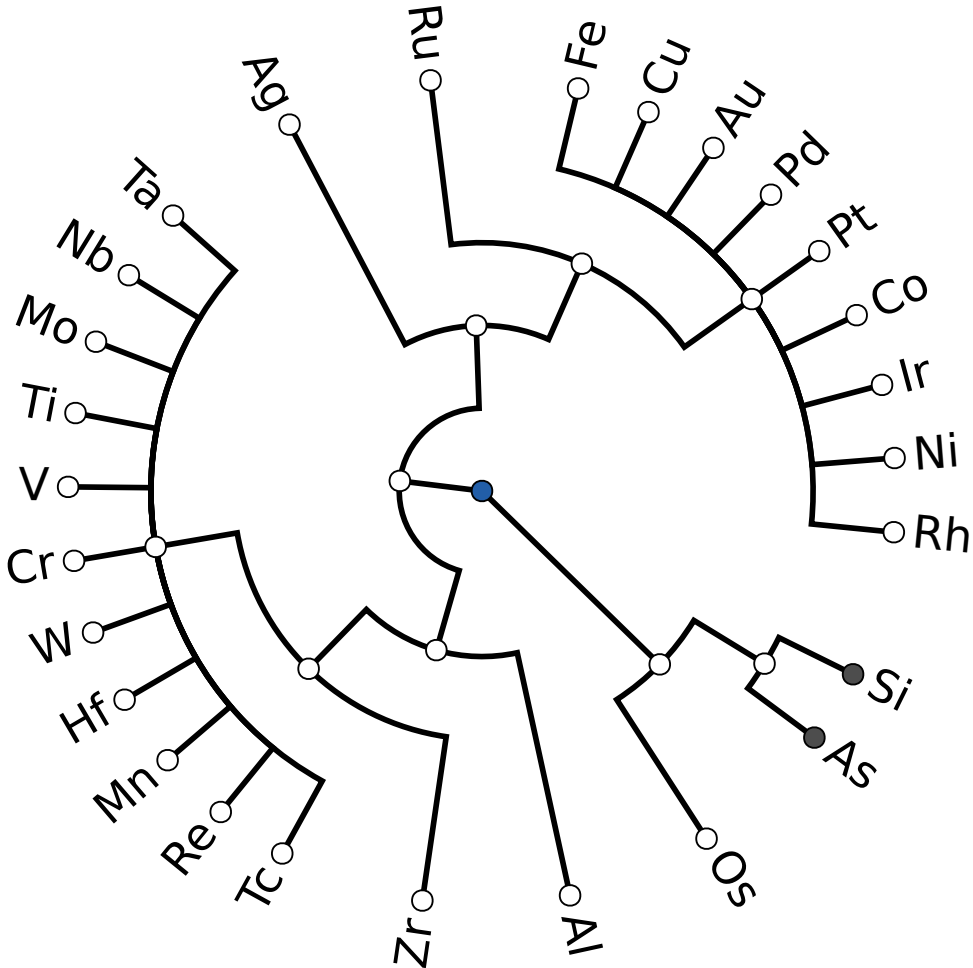

Supplement: Supplementary file 2 — Statistical Source Data. [file 43588_2021_97_MOESM2_ESM.zip › Figure_2/Figure_2f.pdf]

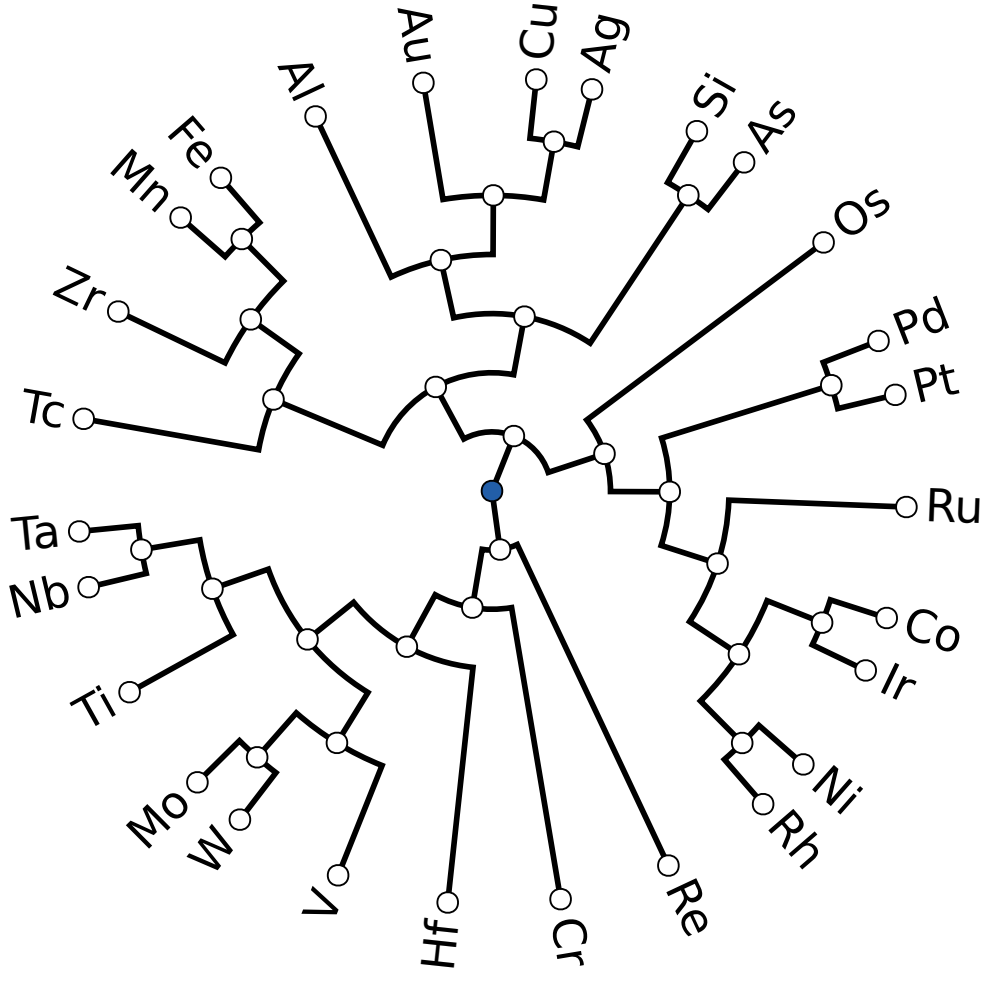

Supplement: Supplementary file 2 — Statistical Source Data. [file 43588_2021_97_MOESM2_ESM.zip › Figure_2/Figure_2h.pdf]

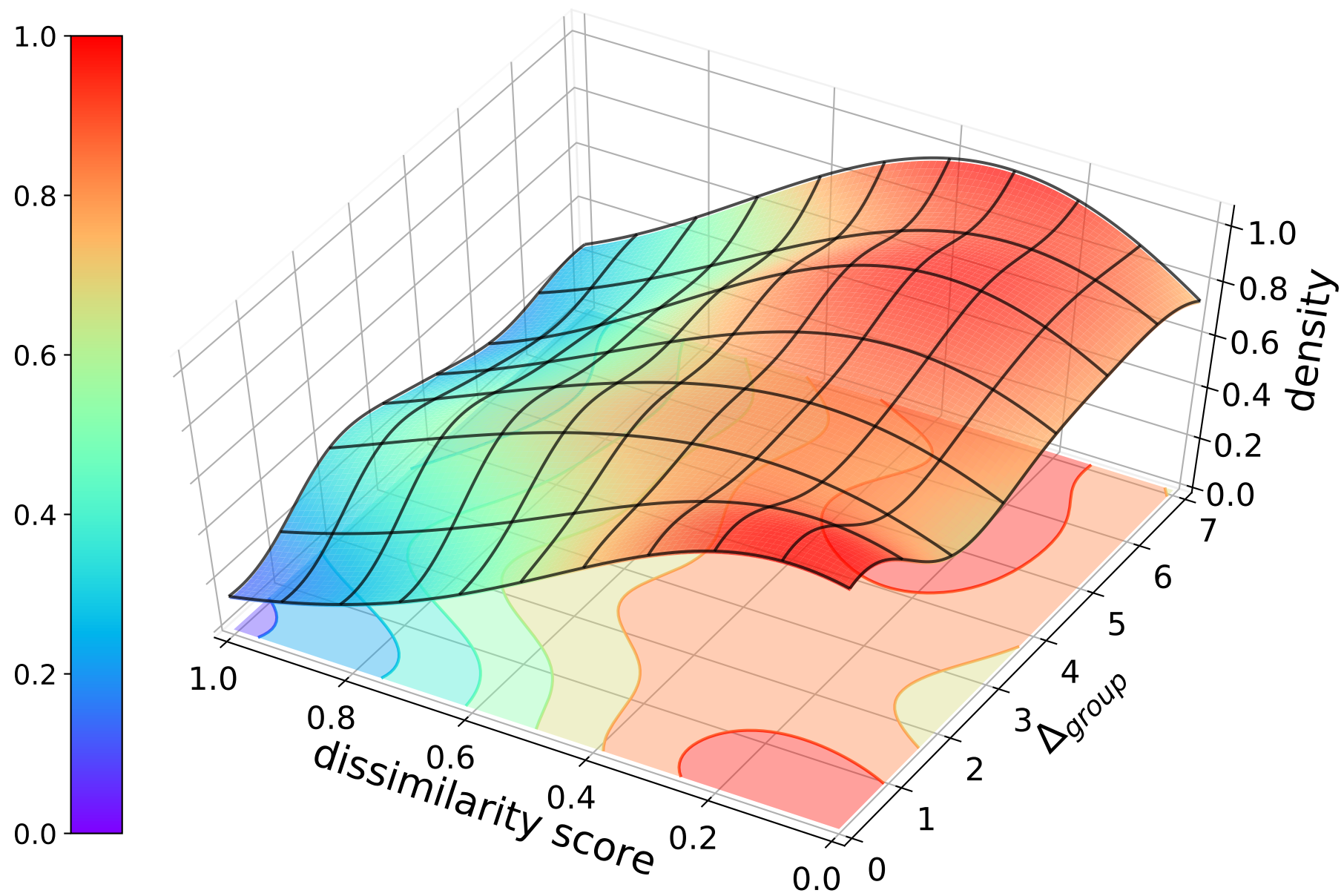

Supplement: Supplementary file 3 — Statistical Source Data. [file 43588_2021_97_MOESM3_ESM.zip › Figure_3/Figure_3d.pdf]

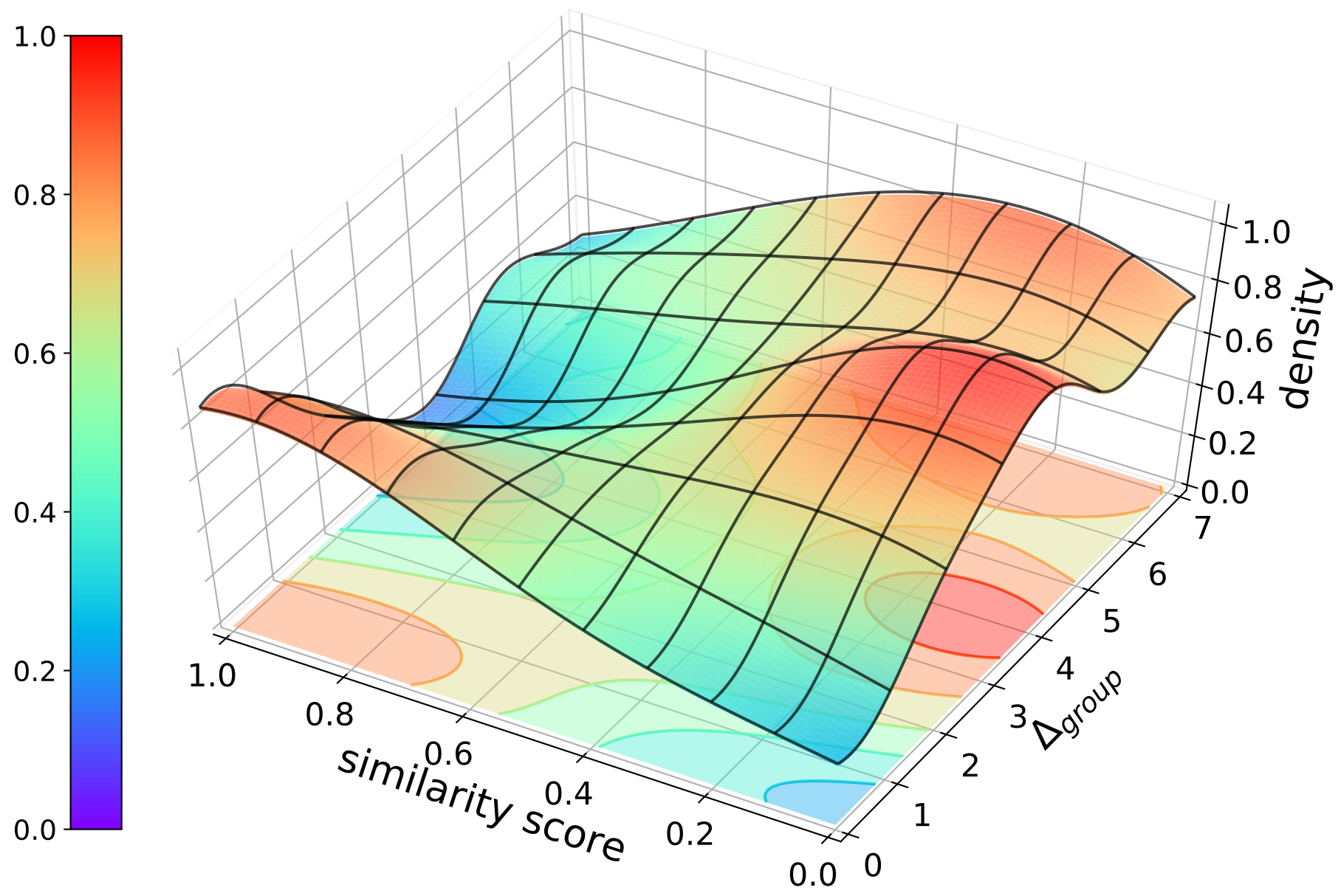

Supplement: Supplementary file 3 — Statistical Source Data. [file 43588_2021_97_MOESM3_ESM.zip › Figure_3/Figure_3a.pdf]

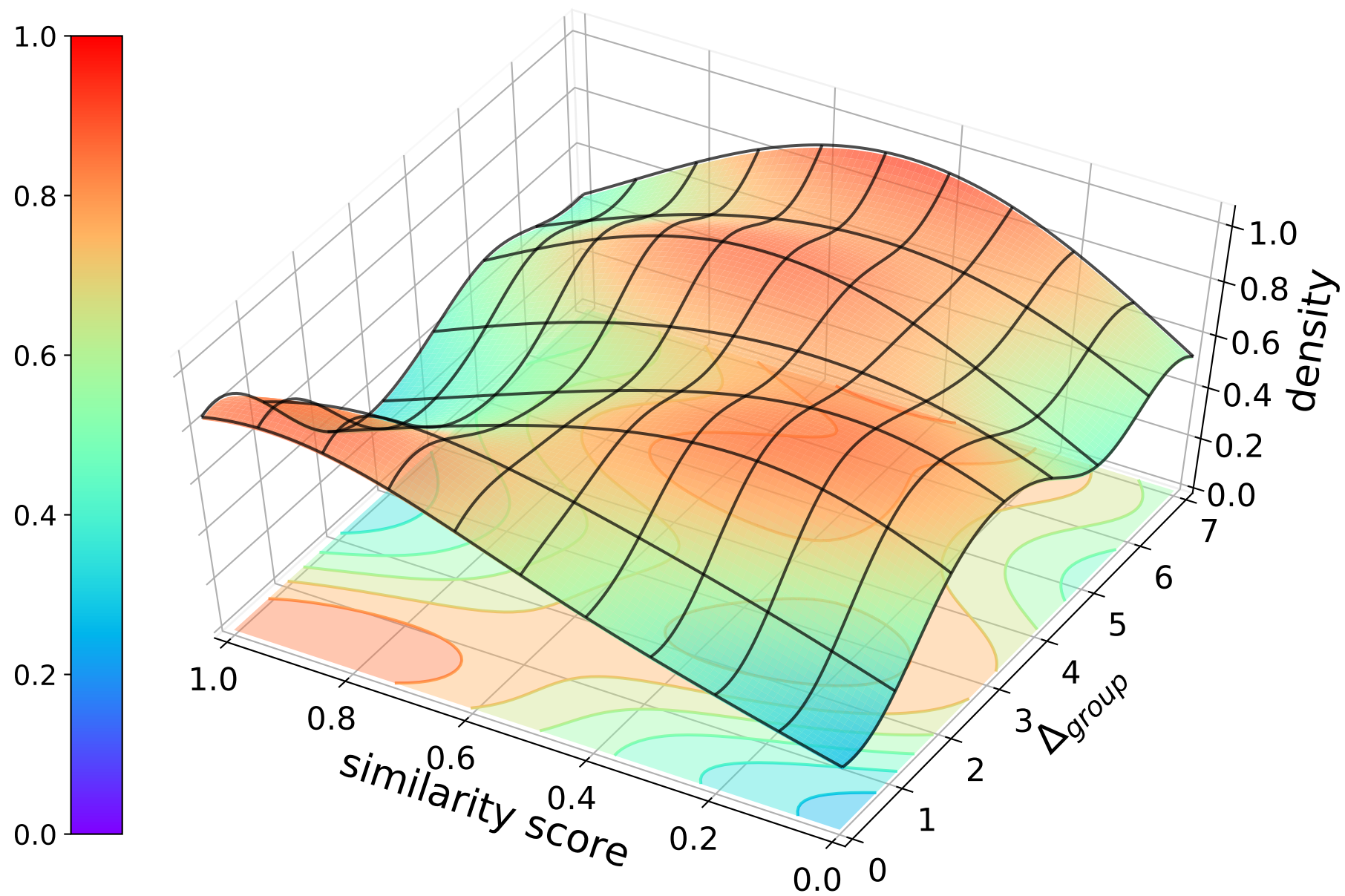

Supplement: Supplementary file 3 — Statistical Source Data. [file 43588_2021_97_MOESM3_ESM.zip › Figure_3/Figure_3c.pdf]

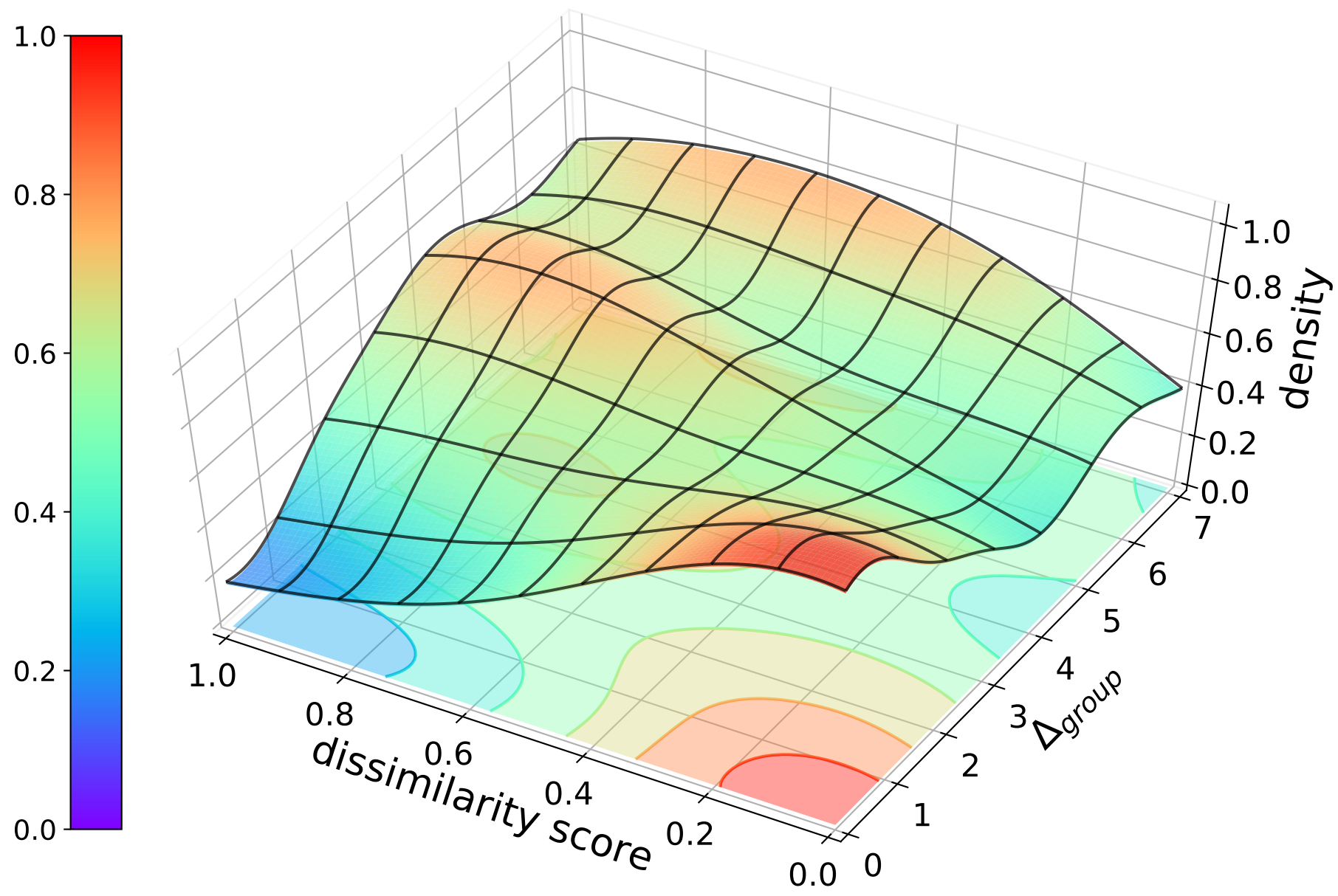

Supplement: Supplementary file 3 — Statistical Source Data. [file 43588_2021_97_MOESM3_ESM.zip › Figure_3/Figure_3b.pdf]

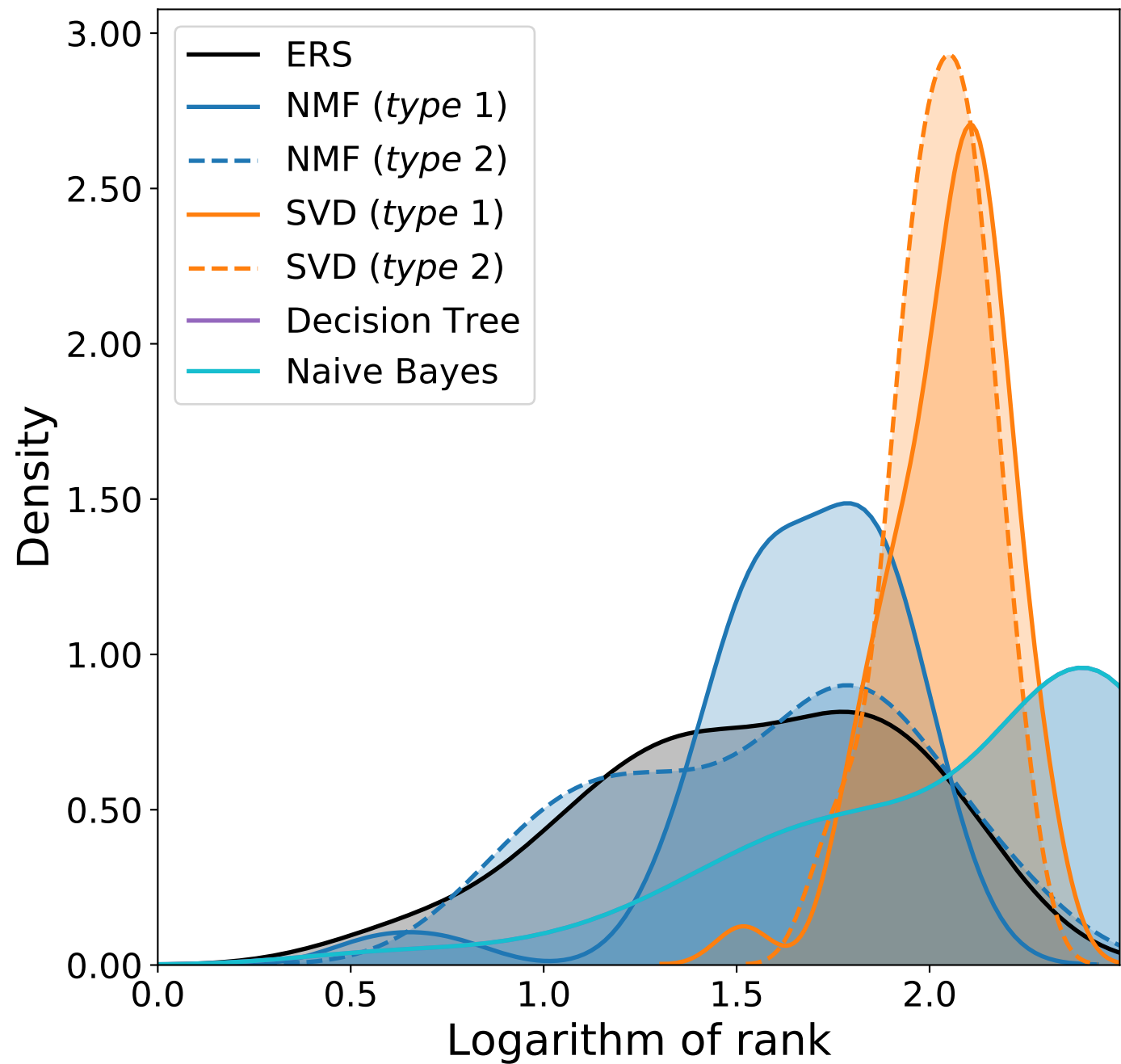

Supplement: Supplementary file 4 — Statistical Source Data. [file 43588_2021_97_MOESM4_ESM.zip › Figure_4/Figure_4a.pdf]

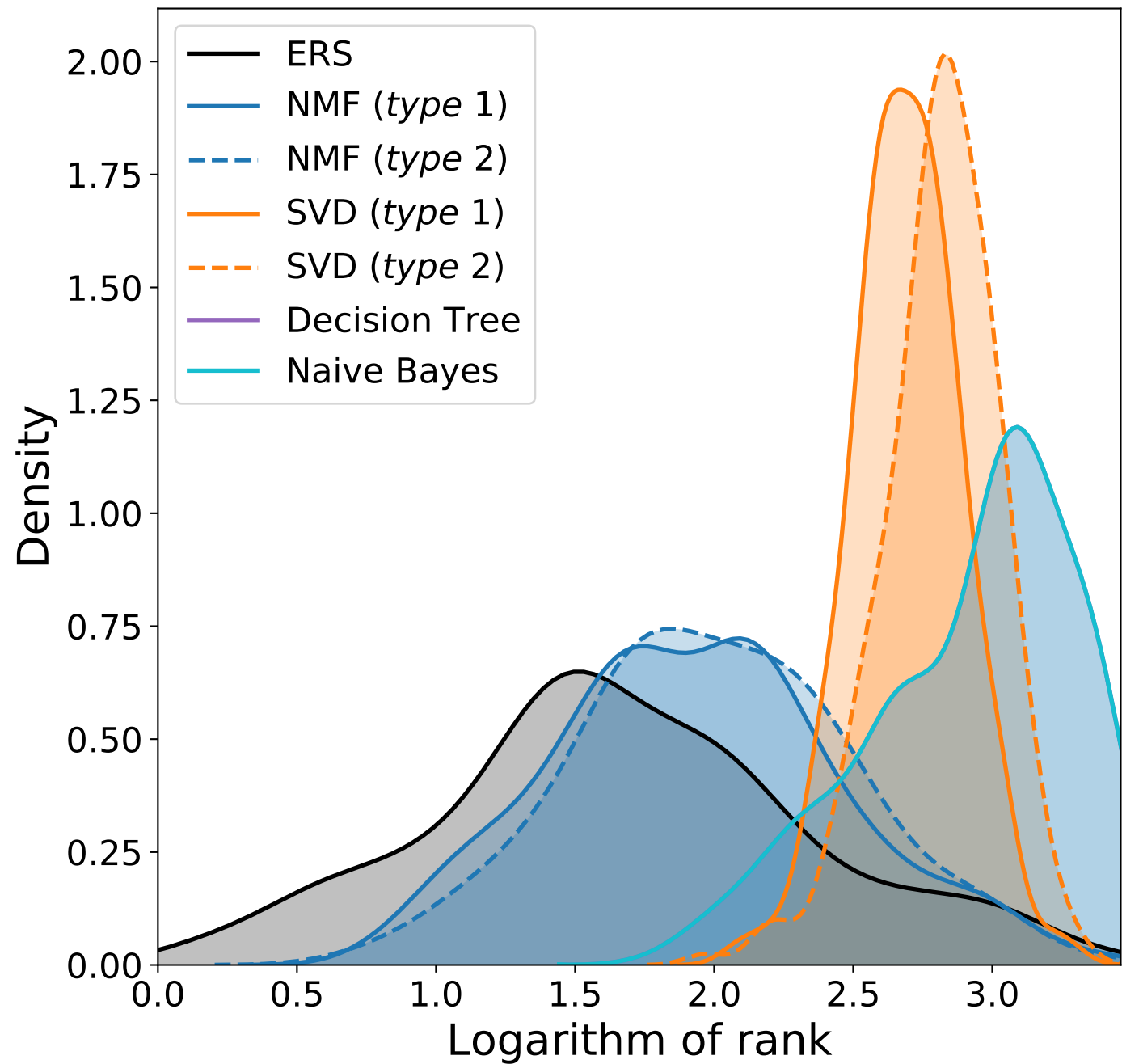

Supplement: Supplementary file 4 — Statistical Source Data. [file 43588_2021_97_MOESM4_ESM.zip › Figure_4/Figure_4b.pdf]

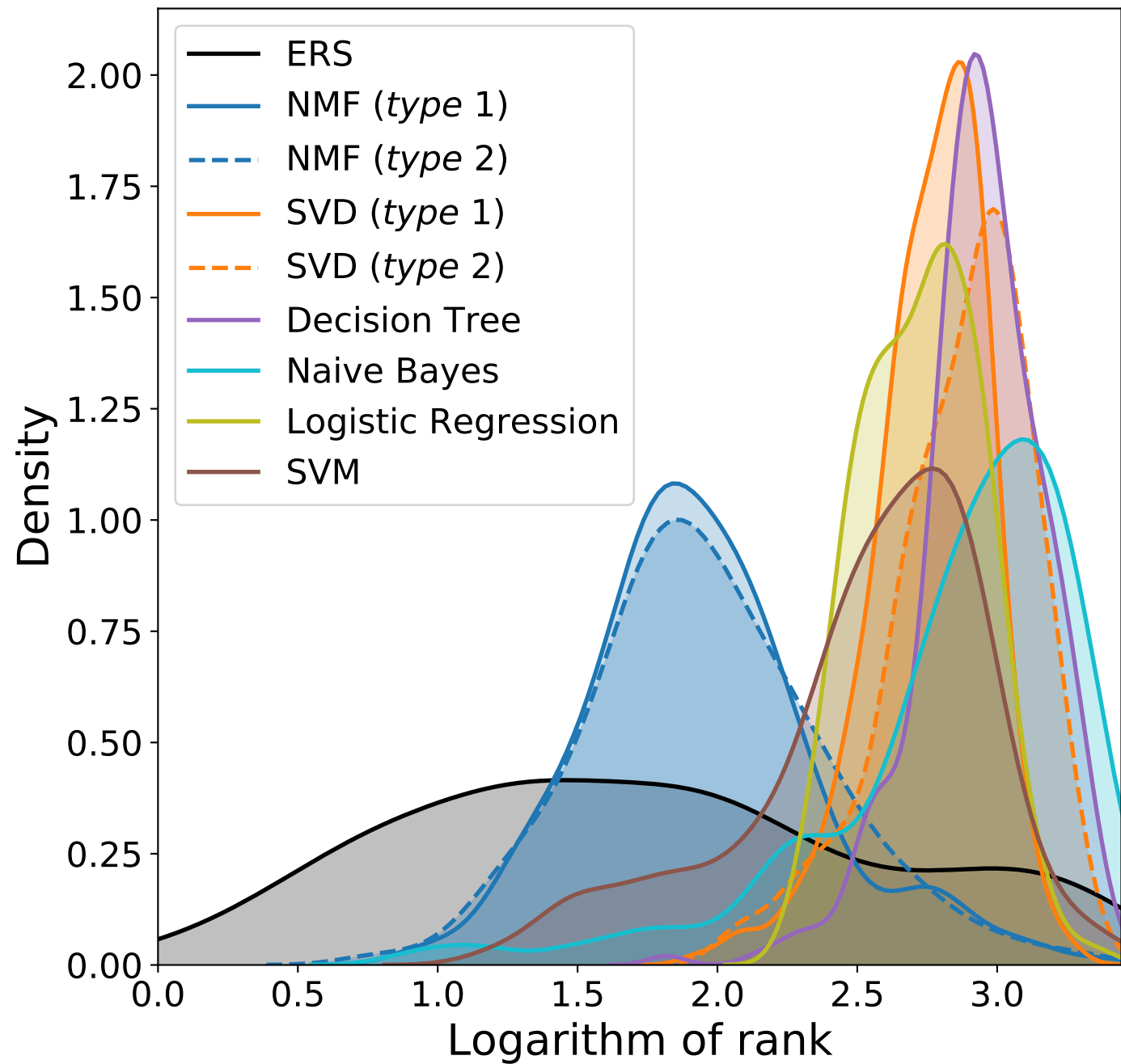

Supplement: Supplementary file 4 — Statistical Source Data. [file 43588_2021_97_MOESM4_ESM.zip › Figure_4/Figure_4c.pdf]

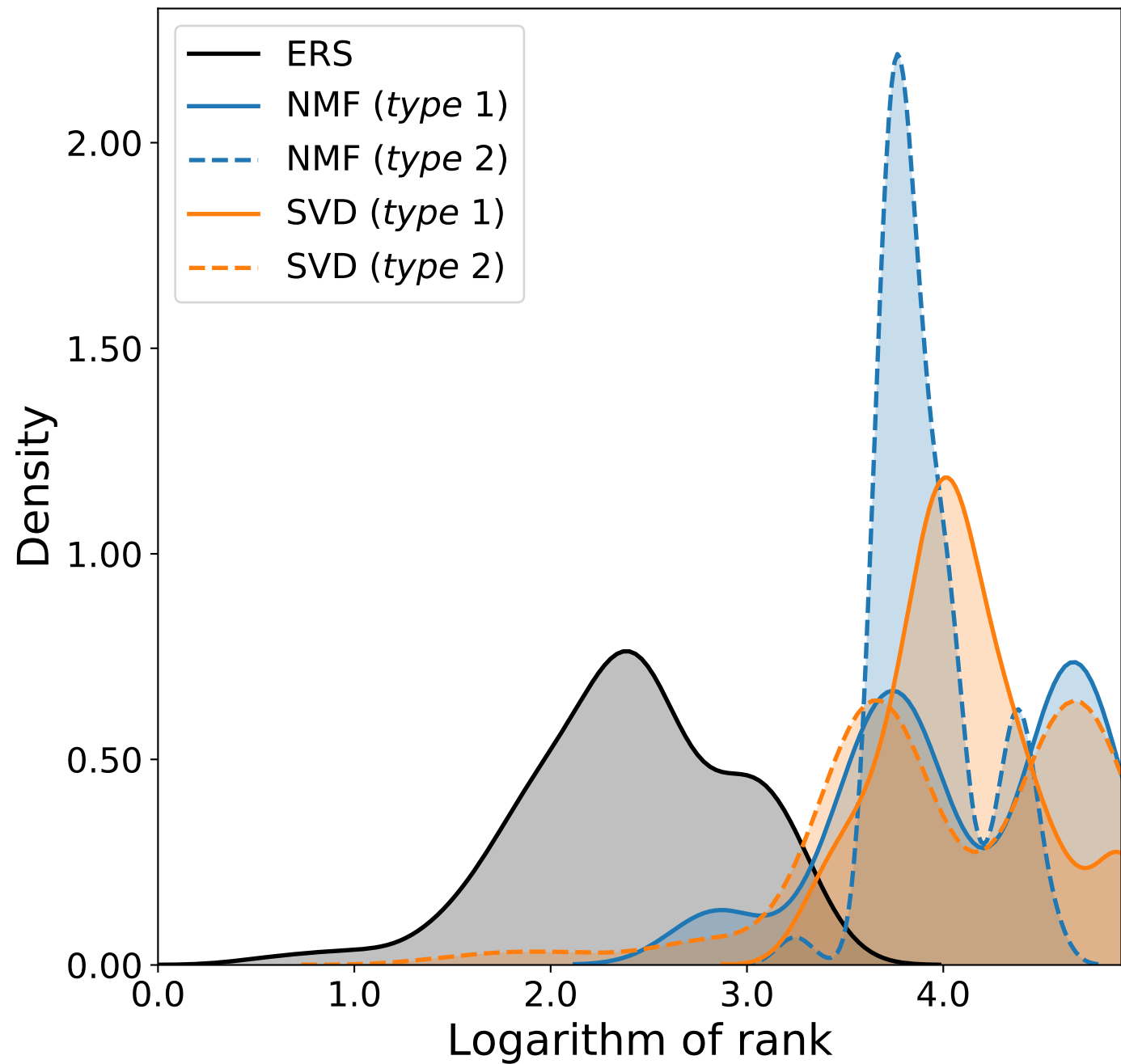

Supplement: Supplementary file 4 — Statistical Source Data. [file 43588_2021_97_MOESM4_ESM.zip › Figure_4/Figure_4g.pdf]

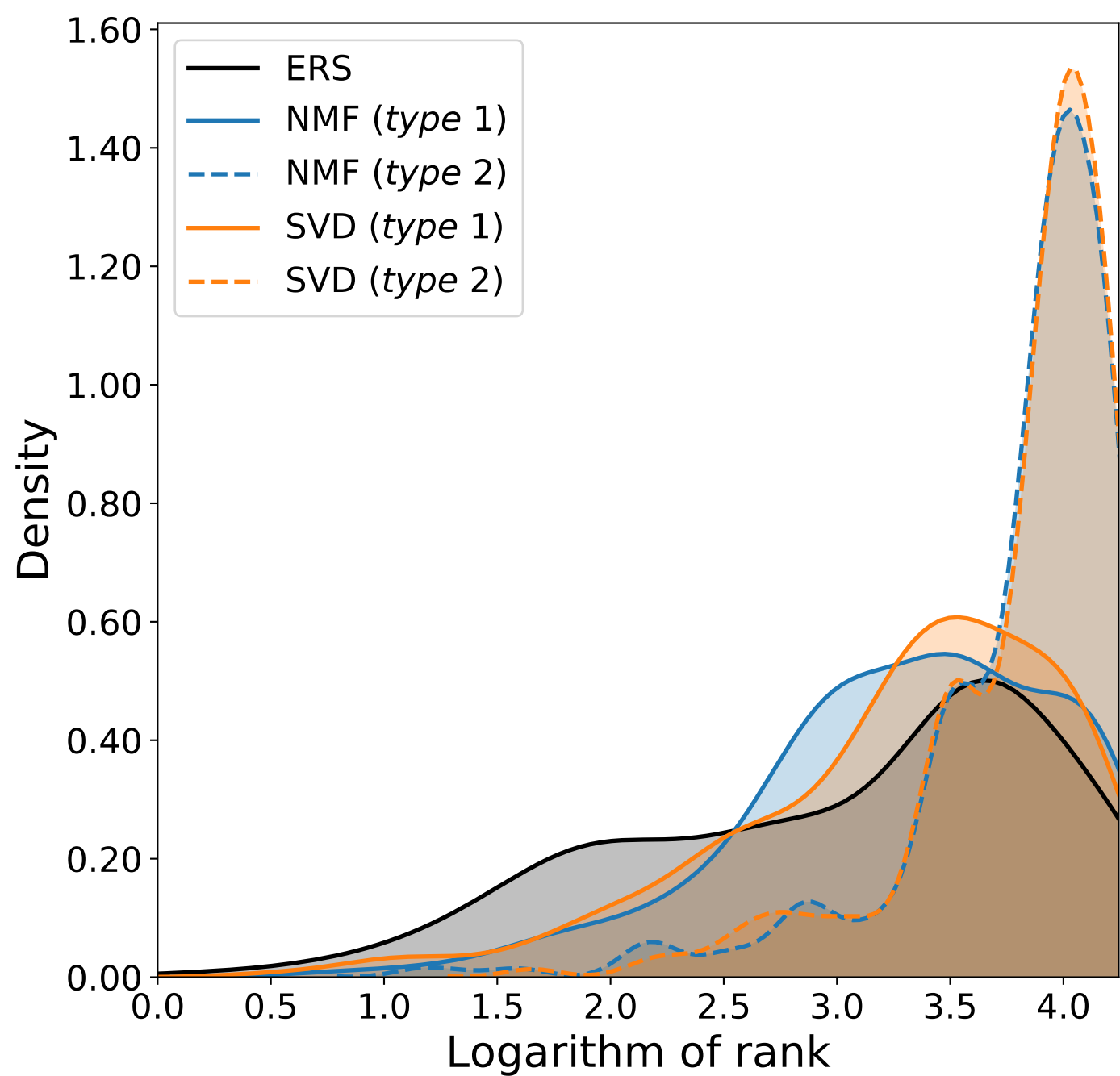

Supplement: Supplementary file 4 — Statistical Source Data. [file 43588_2021_97_MOESM4_ESM.zip › Figure_4/Figure_4f.pdf]

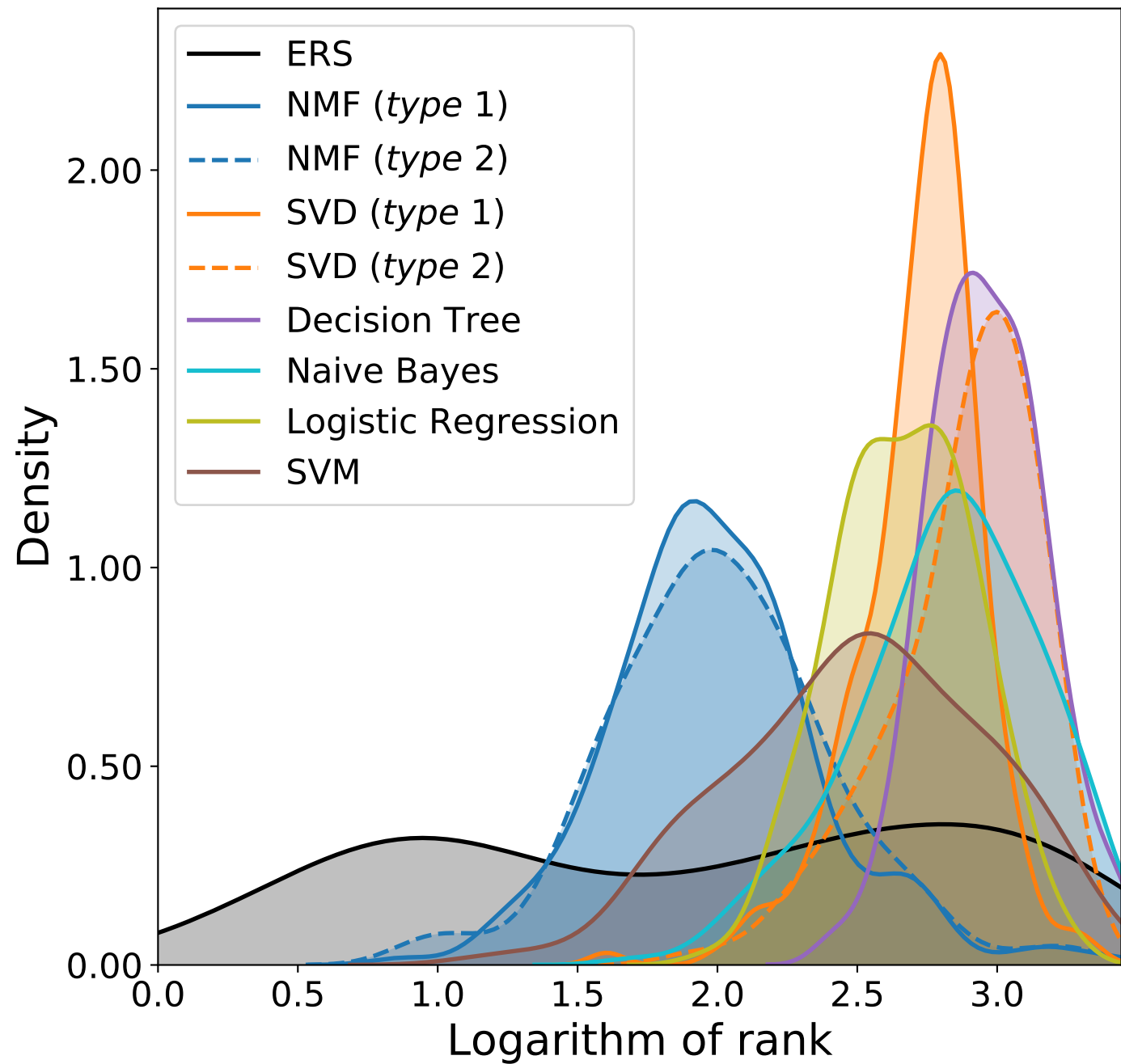

Supplement: Supplementary file 4 — Statistical Source Data. [file 43588_2021_97_MOESM4_ESM.zip › Figure_4/Figure_4d.pdf]

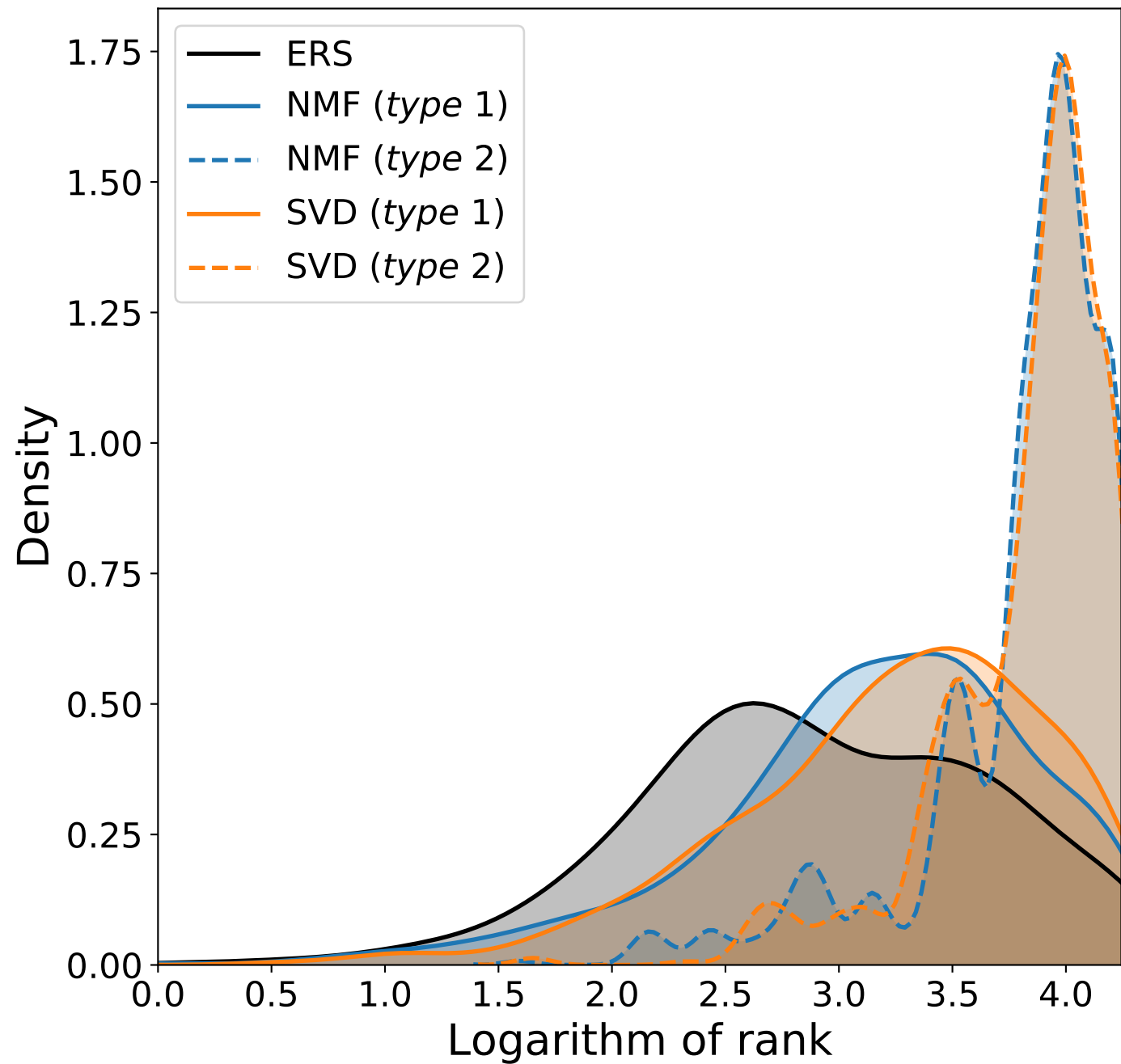

Supplement: Supplementary file 4 — Statistical Source Data. [file 43588_2021_97_MOESM4_ESM.zip › Figure_4/Figure_4e.pdf]

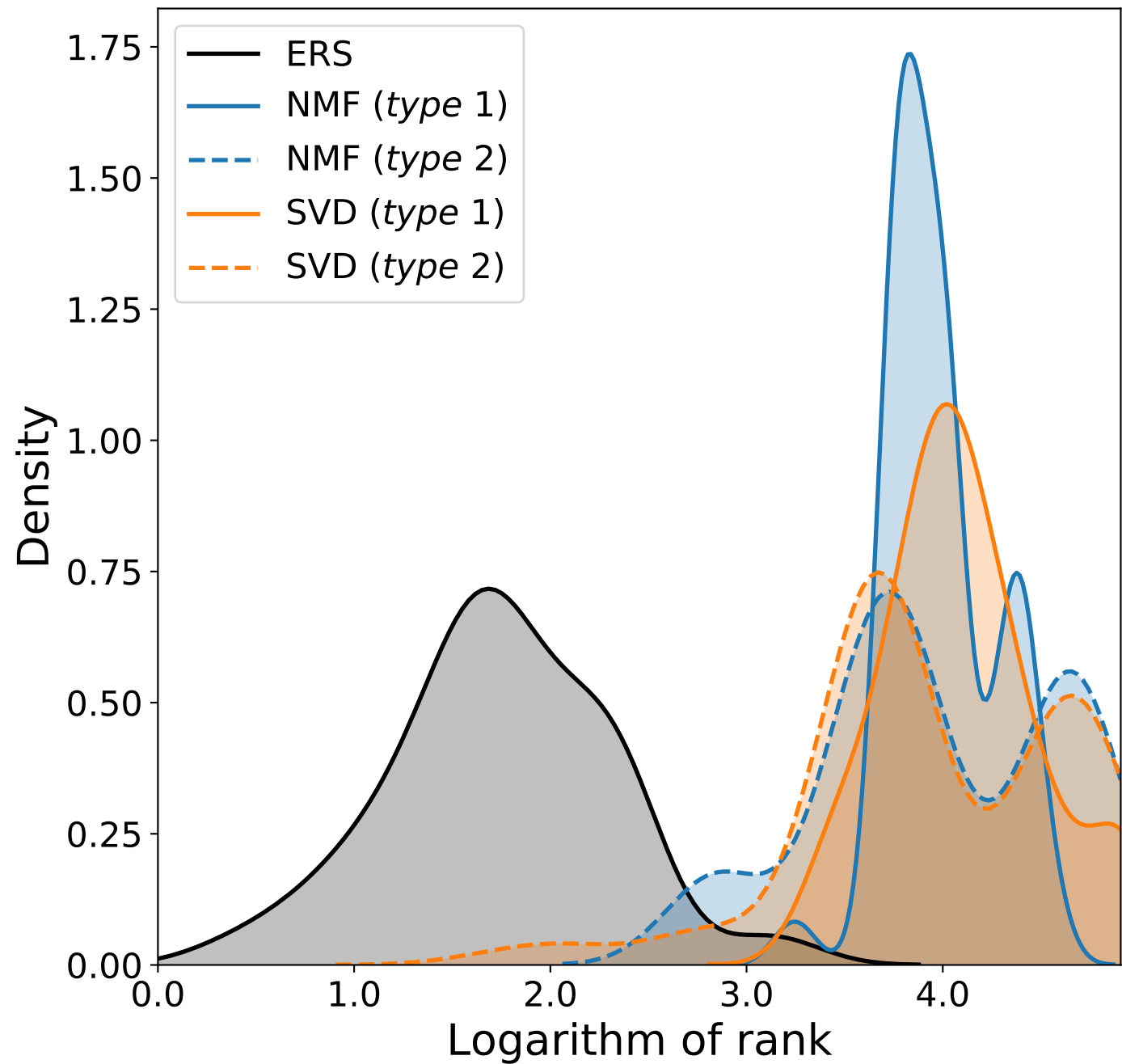

Supplement: Supplementary file 4 — Statistical Source Data. [file 43588_2021_97_MOESM4_ESM.zip › Figure_4/Figure_4h.pdf]

Ni composition, x

0.5  
0.4  
0.3  
0.2  
0.1  
0.0

20

30

40

50

$2\theta$  (deg.)

FCC 111

BCC 110

FCC 200

280

0

Relative intensity (cps)

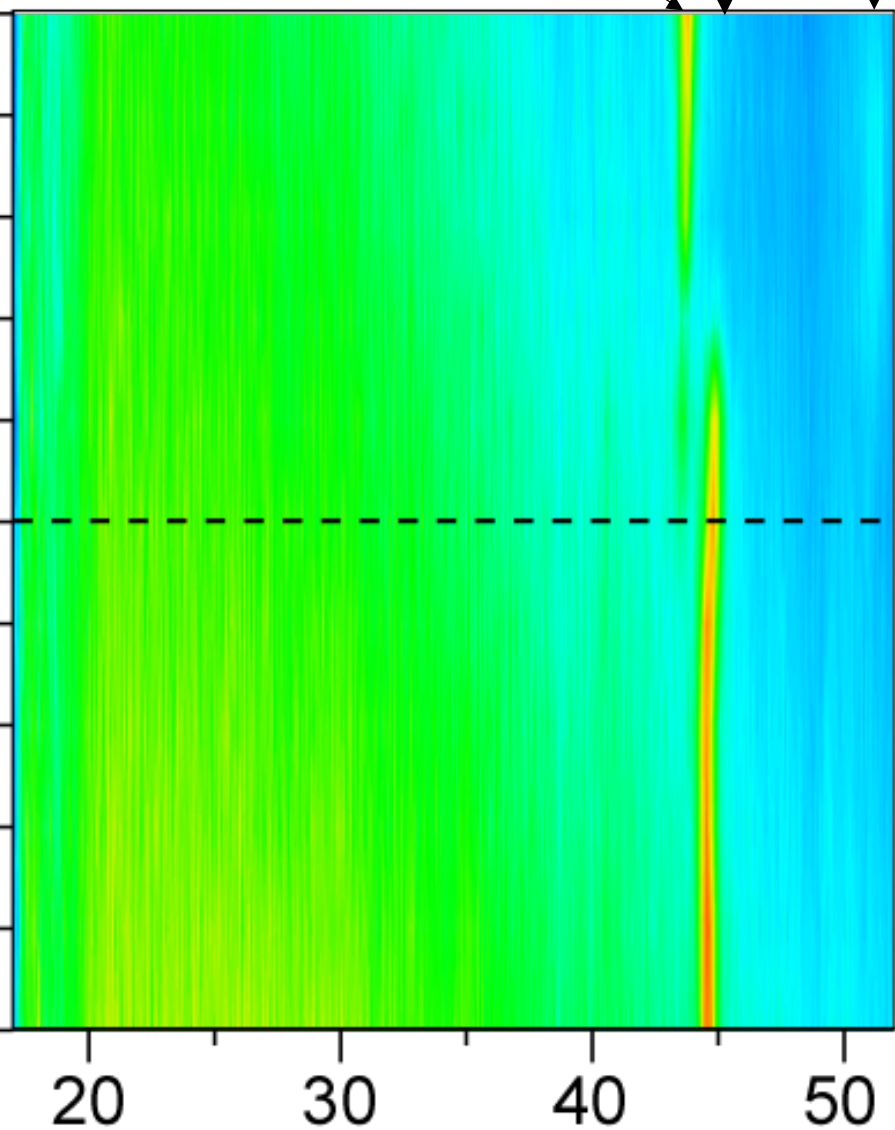

Supplement: Supplementary file 5 — Statistical Source Data and unprocessed figures. [file 43588_2021_97_MOESM5_ESM.zip › Figure_5/Figure_5d.pdf]

$x=0$

$x=0.5$

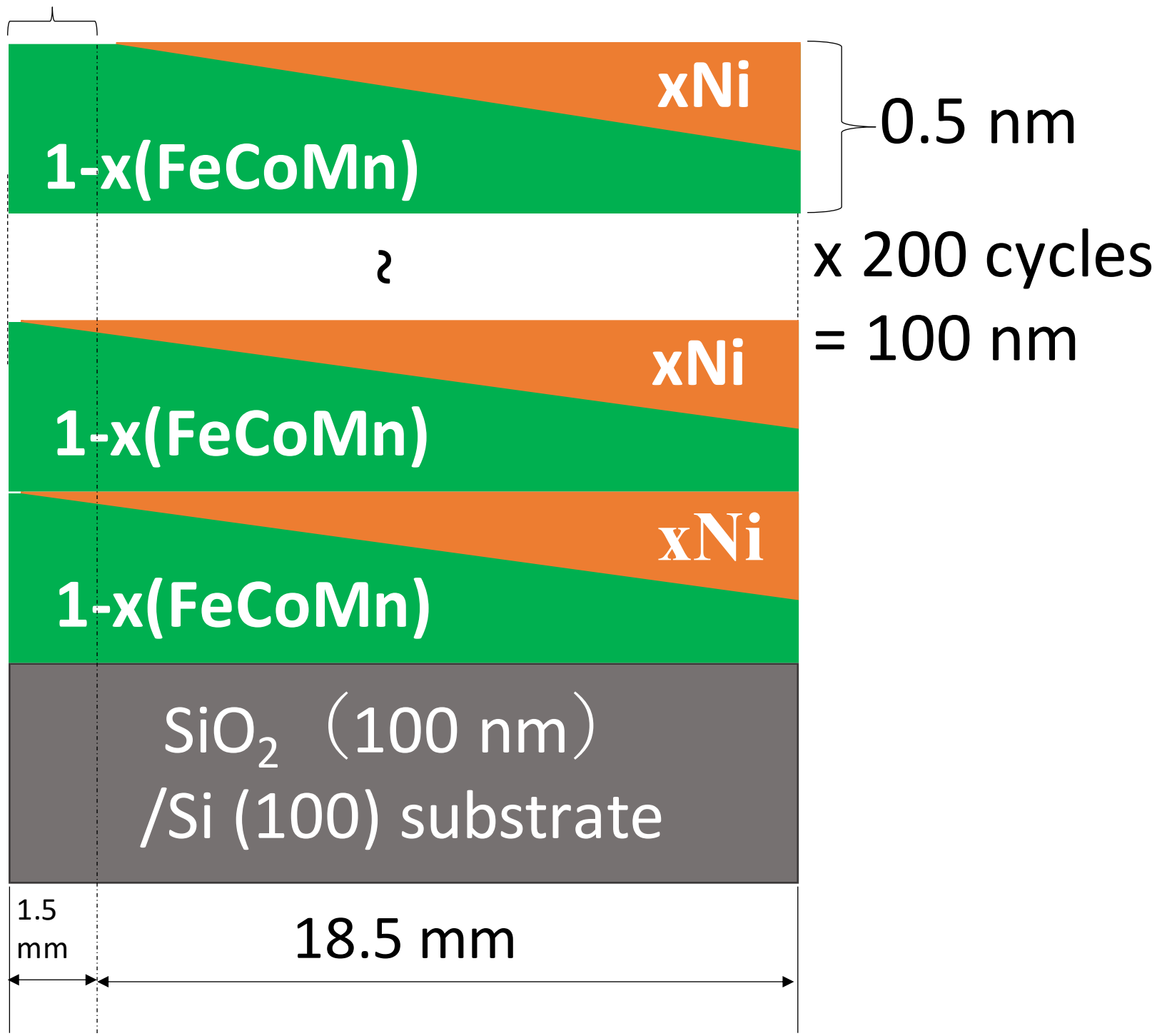

Supplement: Supplementary file 5 — Statistical Source Data and unprocessed figures. [file 43588_2021_97_MOESM5_ESM.zip › Figure_5/Figure_5b.pdf]

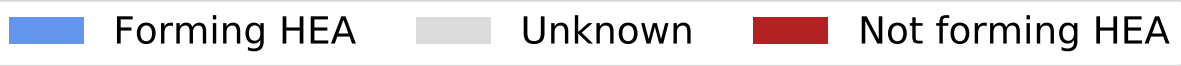

FeMnCoTi

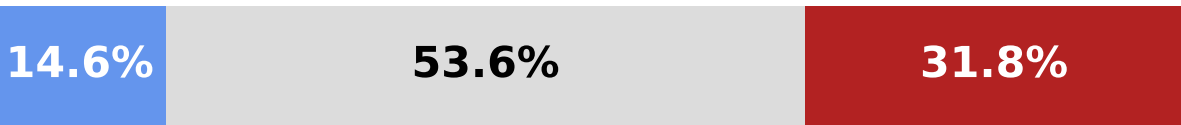

FeMnCoV

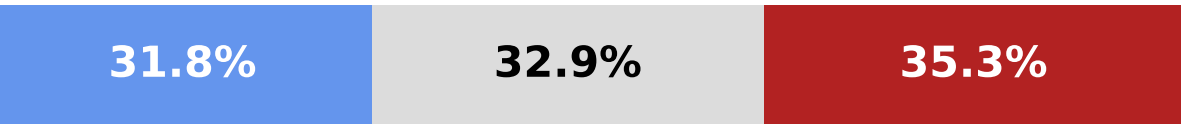

FeMnCoCr

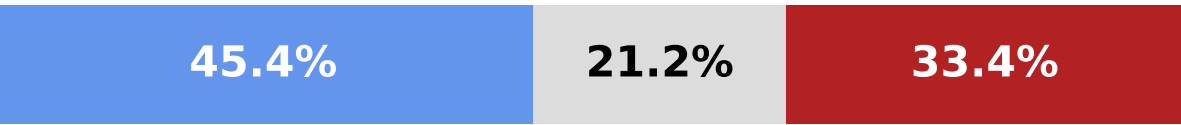

FeMnCoNi

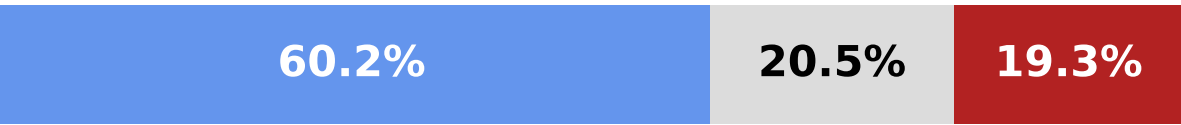

FeMnCoCu

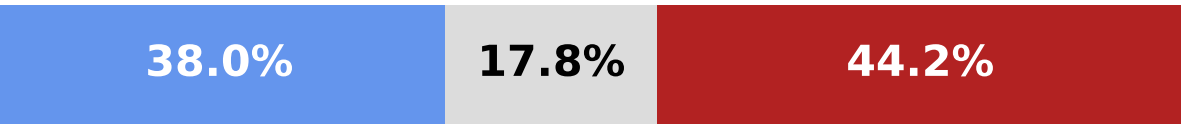

Supplement: Supplementary file 5 — Statistical Source Data and unprocessed figures. [file 43588_2021_97_MOESM5_ESM.zip › Figure_5/Figure_5a.pdf]
