# Supplementary material for: Evidence-based recommender system for high-entropy alloys
Source: Nat Comput Sci. 2021 Jul 19;1(7):470–8. doi: 10.1038/s43588-021-00097-w (PMC10766533; doi:10.1038/s43588-021-00097-w)
Supplement: Supplementary file 5 — Statistical Source Data and unprocessed figures. [file 43588_2021_97_MOESM5_ESM.zip › Figure_5/Figure_5c.pdf]

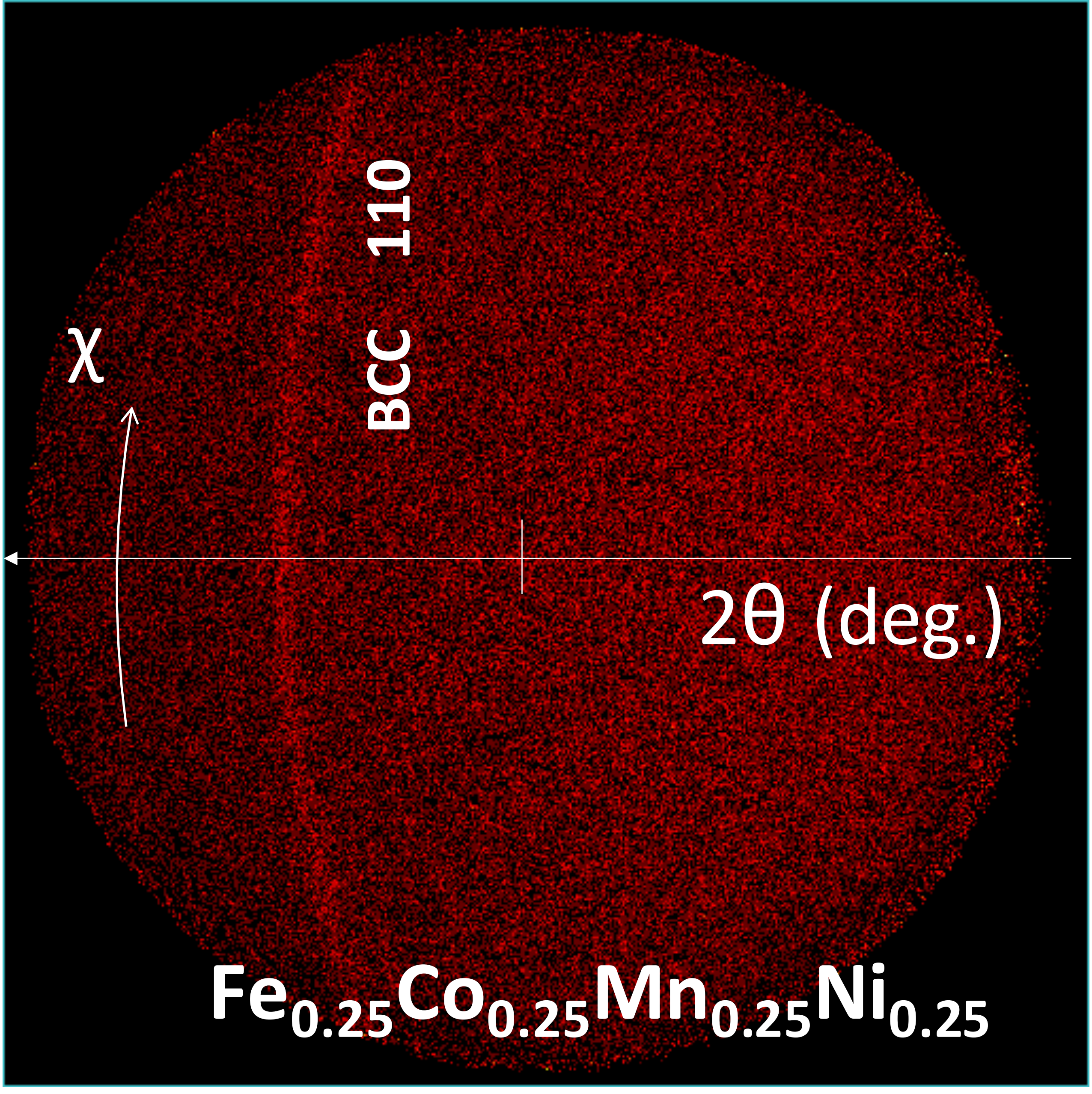
BCC 110
χ
2θ (deg.)
$Fe_{0.25}Co_{0.25}Mn_{0.25}Ni_{0.25}$
